# Current Trends and New Challenges in Marine Phycotoxins

**DOI:** 10.3390/md20030198

**Published:** 2022-03-08

**Authors:** Maria Carmen Louzao, Natalia Vilariño, Carmen Vale, Celia Costas, Alejandro Cao, Sandra Raposo-Garcia, Mercedes R. Vieytes, Luis M. Botana

**Affiliations:** 1 Departamento de Farmacologia, Facultad de Veterinaria, Universidade de Santiago de Compostela, 27002 Lugo, Spain; natalia.vilarino@usc.es (N.V.); mdelcarmen.vale@usc.es (C.V.); celia.costas.sanchez@usc.es (C.C.); alejandro.cao.cancelas@usc.es (A.C.); sandra.raposo.garcia@usc.es (S.R.-G.); 2 Departamento de Fisiologia, Facultad de Veterinaria, Universidade de Santiago de Compostela, 27002 Lugo, Spain; mmercedes.rodriguez@usc.es

**Keywords:** phycotoxin, mechanism of action, toxicity, therapeutic application, detection methods

## Abstract

Marine phycotoxins are a multiplicity of bioactive compounds which are produced by microalgae and bioaccumulate in the marine food web. Phycotoxins affect the ecosystem, pose a threat to human health, and have important economic effects on aquaculture and tourism worldwide. However, human health and food safety have been the primary concerns when considering the impacts of phycotoxins. Phycotoxins toxicity information, often used to set regulatory limits for these toxins in shellfish, lacks traceability of toxicity values highlighting the need for predefined toxicological criteria. Toxicity data together with adequate detection methods for monitoring procedures are crucial to protect human health. However, despite technological advances, there are still methodological uncertainties and high demand for universal phycotoxin detectors. This review focuses on these topics, including uncertainties of climate change, providing an overview of the current information as well as future perspectives.

## 1. Introduction

Harmful algal blooms (HABs) are a significant problem in coastal waters, particularly when they produce phycotoxins that accumulate in shellfish or fish, leading to the poisoning of humans and animals. The species that cause HABs are diverse, as are the habitats in which they occur. Climate change could affect the prevalence of HABs and impact of phycotoxins on human and ecosystem health [1]. Phycotoxins cause human intoxications with clinical symptoms ranging from intestinal to neurological effects but may also provoke respiratory distress, dermatological problems, or even death [2]. There are currently six main phycotoxin poisoning syndromes that can occur after ingestion of contaminated shellfish, fish, or fishery products, which are paralytic, neurotoxic, amnesic, diarrhetic, and azaspiracid shellfish poisoning (PSP, NSP, ASP, DSP, and AZP) and ciguatera fish poisoning (CFP). However, potential seafood contamination with tetrodotoxin or palytoxin is also of concern. Additionally, phycotoxins such as pectenotoxins, yessotoxins, and the cyclic imines are also considered.

**Figure 1:**
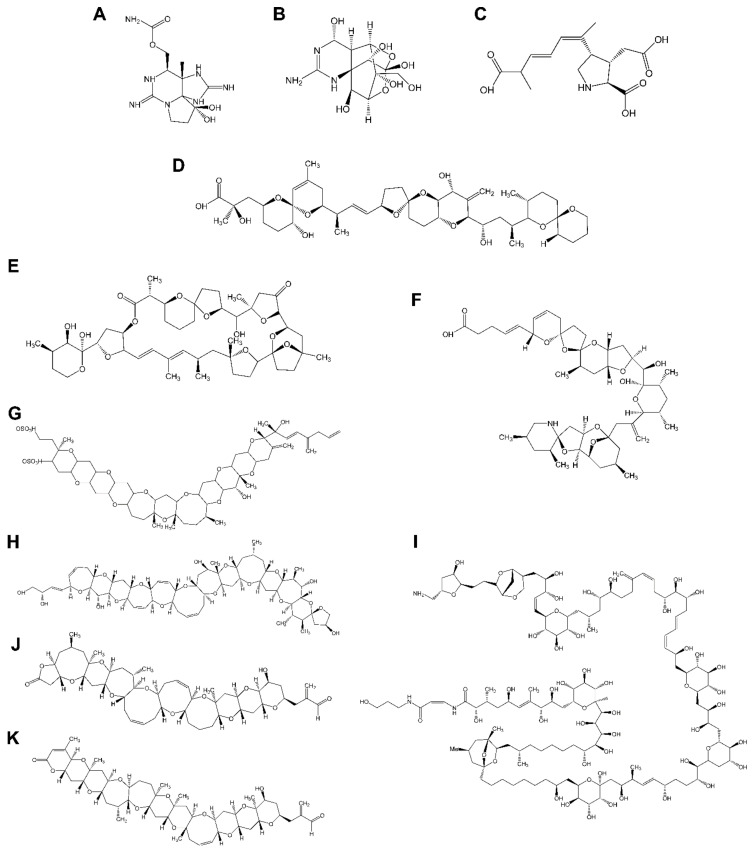
Chemical structure of the representative compounds of each group of toxins mentioned in the text: saxitoxin (**A**), tetrodotoxin (**B**), domoic acid (**C**), okadaic acid (**D**), pectenotoxin 2 (**E**), azaspiracid 1 (**F**), yessotoxin (**G**), Pacific ciguatoxin 1 (**H**), palytoxin (**I**), brevetoxin 1 (**J**), and brevetoxin 2 (**K**).

Paralytic shellfish poisoning (PSP) has been widely reported in many parts of the world [3]. Paralytic shellfish toxins (PSTs) include saxitoxin (Figure 1A) and its analogues neurotoxic alkaloids produced mainly by dinoflagellates of the genera *Alexandrium*, by the species *Pyrodinium bahamense* and *Gymnodinium catenatum* and by benthic and planktonic marine cyanobacteria such as *Anabaena*, *Cylindrospermopsis, Aphanizomenon*, *Planktothrix,* and *Lymgbya*. PSTs act through the reversible blockade of the voltage-gated sodium channels in excitable membranes compromising the propagation of neural impulses in peripheral nerves and skeletal muscles (Figure 1) [4,5,6]. PSTs share a similar molecular weight, toxicity, and mechanism of action with tetrodotoxin (TTX) (Figure 1B) [7]. The main exposure to TTX for humans comes from fish from the Tetraodontidae family (pufferfish), which is forbidden in the European market, but this toxin also occurs in marine gastropods, oysters, mussels, and fish other than pufferfish (See Katikou et al. 2022 for updated review of TTX) [8].

Amnesic shellfish poisoning (ASP) is caused by domoic acid (DA) (Figure 1C). This potent natural toxin is produced by the diatoms *Nitzschia*, *Pseudonitzschia*, and *Amphora* and found worldwide [9]. Filter feeding marine life, such as clams, oysters, mussels, and crabs, can accumulate DA and pass the toxin to humans and wildlife [10].

Diarrhetic shellfish poisoning (DSP) causes symptoms of gastrointestinal dysfunction, such as nausea, vomiting, and diarrhea, but it is not considered lethal. Diarrhetic shellfish toxins (DSTs) include okadaic acid (OA) (Figure 1D) and dinophysistoxins (DTXs), lipophilic molecules that are among the most prevalent marine toxins in Europe and Asia or South America [11,12]. DSTs are produced by marine dinoflagellates belonging to the genera *Dinophysis* and *Prorocentrum* [13,14]. Among the group of lipophilic phycotoxins, other families have been described: pectenotoxins (PTXs) (Figure 1E), yessotoxins (YTXs) (Figure 1G), and cyclic imines (pinnatoxins (PnTXs), spirolides (SPXs), gymnodimines (GYMs), pteriatoxins (PtTXs), porocentrolides (PcTXs), spiro-prorocentrimines, and portimine). The potent neurotoxicity of cyclic imines led to concerns about their potential threat to shellfish consumers [15].

Azaspiracid shellfish poisoning (AZP) (Figure 1F) is a toxicity syndrome in humans due to the ingestion of azaspiracid-bearing shellfish and causes mainly gastrointestinal symptoms [16,17]. Therefore, azaspiracids (AZAs) were initially included in the DSP group. The characterization of their chemical structure, along with a different mechanism of action, led to their classification as a stand-alone group [18,19]. AZAs are produced by planktonic species of the genus *Azadinium* and *Amphidoma* and can be further biotransformed by accumulating shellfish [20,21]. They were reported in mollusks and crustacean species from numerous European countries [22,23,24] as well as in shellfish from Africa, Asia, and America [25].

**Figure 2:**
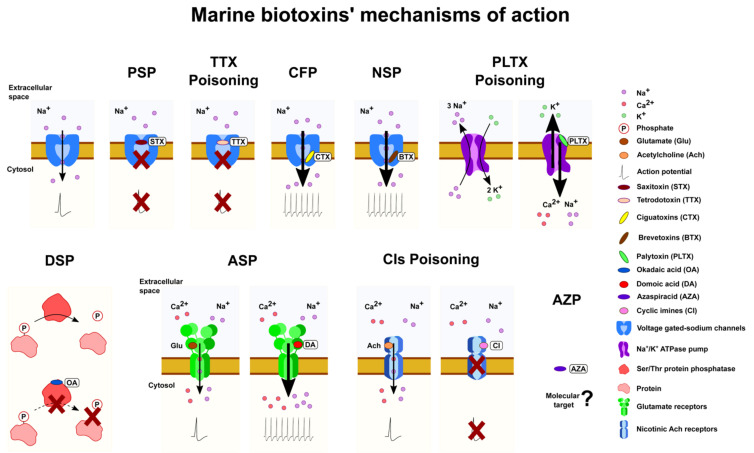
Mechanisms of action of marine biotoxins. Saxitoxin (STX) and tetrodotoxin (TTX) bind to voltage gated sodium channels (VGSCs) site 1, blocking Na^+^ flux. Consequently, action potential transmission is suppressed in neurons and muscles, resulting in paralytic shellfish poisoning (PSP) and TTX poisoning, respectively. Ciguatoxins (CTXs) and Brevetoxins (BTXs) bind to VGSCs site 5, keeping the channel in an open state. Increased Na^+^ influx triggers action potential in excitable cells, leading to Ciguatera and Neurotoxic Shellfish Poisoning (NSP), respectively. Palytoxins (PLTXs) block the active transport of Na^+^ and K^+^ by Na^+^/K^+^ ATPase, converting the pump in a non-selective cation channel. The ion imbalance results in the wide symptomatology of PLTX poisoning. Okadaic acid (OA) is known to inhibit Ser/Thr protein phosphatases, resulting in proteins’ hyperphosphorylation. However, its relationship with the symptomatology developed and, thus, diarrhetic shellfish poisoning (DSP) is unclear. Domoic acid (DA) bind to kainate and AMPA receptors in neurons inducing cells overexcitability. As a result, amnesic shellfish poisoning (ASP) is developed. Cyclic imines (CIs) are competitive antagonists of nicotinic acetylcholine receptors (nAChRs). Therefore, they reversely block ACh stimulated muscle contraction. The azaspiracids (AZAs) molecular pathway leading to AZAs shellfish poisoning (AZP) remains unknown.

Ciguatera fish poisoning or Ciguatera is the most common non-bacterial human illness associated with seafood consumption across the globe affecting between 50,000 and 500,000 people annually including rare lethal cases [26,27,28]. Ciguatoxins (CTXs) (Figure 1H) are lipid soluble, thermally-stable toxins responsible for Ciguatera and produced by microalgae within the *Gambierdiscus* and *Fukuyoa* genus. *Gambierdiscus* species also synthesize other toxins, including gambieric acids, gambierol, gambierone, gambieroxides, and maitotoxins [29]. However, until now there has been no evidence of their involvement in human Ciguatera cases [30]. Ciguatoxins are chemically similar to brevetoxins (Figure 1J and K) and both act on voltage-gated sodium channels by binding to receptor site 5, causing depolarization of neuronal and muscle cell membranes and triggering human and animal neurological symptoms (Figure 2) [31,32]. Brevetoxins (BTXs) are a group of polyether-toxins that cause neurotoxic shellfish poisoning (NSP) named after the more or less pronounced neurological symptoms that co-occur with gastrointestinal signs. Humans are exposed to brevetoxins through the ingestion of shellfish in which the toxins seem not to have any adverse effects. The aerosol containing the toxin also induces non-fatal effects on human health including skin irritation, non-productive cough, shortness of breath, and tearing. There has been only a small number of sporadic cases of NSP in humans, with hospitalization but no fatalities. However, brevetoxins have been implicated in the death of large numbers of fish and in morbidity and mortality of marine mammals. To date, no cases of NSP have been reported in Europe [33]; however, the presence of these toxins in shellfish from the Mediterranean Sea [34] raises the question of potential emergence of this group of toxins in areas preserved until now (see Hort et al. 2021 for an updated review of BTX) [35].

Palytoxin (PLTX) (Figure 1I) is a very potent natural toxin responsible for seafood poisoning and produced by soft corals of the genera *Palythoa*, *Zoanthus*, and *Parazoanthus*, by planktonic and benthic dinoflagellates of the genus *Ostreopsis*, and by cyanobacteria of the genus *Trichodesmium*. In recent decades, species of the genus *Ostreopsis* are proliferating in temperate latitudes including the Mediterranean Sea Coast where recurrent blooms have occurred [36,37]. Even though PLTXs are not regulated in seafood in Europe, these toxins have been shown to be harmful and occur in European coasts.

This review is focused on the toxins mentioned above and organized in four major parts: The first part addresses the lack of traceability of toxicity values, the second part discusses the mechanisms of action and toxicity including the need to establish objective toxicity parameters, the third part addresses marine toxins as a source of drugs, and the fourth part includes an update on toxin detection methods update and the need for universal detectors and ends with climate change uncertainties.

## 2. Lack of Traceability of Toxicity Values

During the last decades, the occurrence and intensity of marine biotoxins intoxications were boosted paralleling a worldwide increase in harmful algae bloom (HAB) events due to international trade expansion and anthropogenic eutrophication [38]. In this situation, the need to obtain data about the toxicity of the different marine biotoxins arises in order to set safety regulatory limits for these compounds in seafood and protect the health of consumers. Traditionally, the main assays employed to evaluate the toxicity of marine biotoxins were the in vivo mousse bioassay (MBA) and in vitro cell viability assays [11]. However, the lack of certified reference materials and the lack of standard operating procedures have hampered the obtention of reliable data regarding toxicity.

The problem arises when the assay method is not suitable for the evaluation of the real toxicity of the toxins. A representative example of this problem is that the reference to estimate the relative potency of some toxins that are not considered lethal is the MBA. This assay considers the death of the mice after intraperitoneal administration of the compounds, though the main consequence of most of these intoxications is not death but the reported acute symptoms after oral or epidermal exposure mentioned above [39,40,41] and long-term sequelae for some toxins, as is the case for CTXs [42]. There is scarce information about the impacts of long-term low-level exposure [41,43,44,45]. These types of studies are necessary to re-evaluate the real risk of the exposure of humans to these toxins since the main health risk for humans is a chronic exposure to low levels of these toxins.

In other cases, the use of cell-based assays may be controversial since not all the cell lines have the same characteristics. In vitro data are particularly useful when the mechanism of action of the toxin is known; however, in toxins which the mechanism of action remains unknown, it is difficult to select a proper cell line for these studies. A representative example of this issue is that the reference cell line to detect and determine ciguatoxin toxicity is a neuroblastoma cell line. The main cellular targets of CTXs are voltage gated sodium channels (VGSCs), and undifferentiated neuroblastoma cells express only Na_v_1.7 (most prominent) and Na_v_1.3 sodium channels, while the other sodium channels are expressed at low levels in cells cultured up to 20 passages [46]. However, current assays for the evaluation of ciguatoxins effects in neuroblastoma cell lines use cells cultured up to passages 383 to 810 [47].

Another source of controversy is the purity of the compounds used in the toxicity assays. Certified reference materials are not available for most of the marine biotoxins; thus, it is difficult to really know the purity and the quantity of the toxin used if they were not determined using analytical methods [48]. The route of administration of the compounds is also a source of discrepancy since many toxicological data are based on the administration of the toxins through the intraperitoneal route, which is less relevant for the evaluation of human exposure than the oral route [48].

The limits for marine biotoxins for international trade were set over a decade ago by the Codex Committee on Fish and Fishery Products (CCFFP), that has developed the standard for Live and raw bivalve mollusks (CODEX STAN 292-2008) adopted in 2008, amended in 2013 and revised in 2014 and 2015 [49]. This standard identifies maximum permissible levels in mollusks flesh for five groups of marine toxins. Specifically, the maximum levels for toxins with neurotoxic activity were established as follows: 0.8 mg for saxitoxin (STX)/kg, 20 mg/kg for domoic acid (DA), 200 mouse units or eq/kg for brevetoxins (BTX), and 0.16 mg/kg for azaspiracids (AZA). It is noteworthy that each group of toxins comprises many analogues, and even then, the limits of toxins are represented according to the total toxicity of the analogues, which is not proved in a standardized form. Traditionally, regulatory limits were established using the mousse bioassay, which involves the intraperitoneal administration of seafood extracts to mice [50], thus providing information about the total toxicity of the sample. The advances in technologies together with changes in legislation and ethical concerns have allowed to increase the number of alternative methods to MBA for toxin monitoring purposes. Any alternative method should provide an equivalent level of protection to consumers as the techniques used as reference and should be interlaboratory validated through international systems such as the Association of Official Analytical Chemists (AOAC) or the European Committee for Normalization (CEN) [51]. Single laboratory validation is only acceptable to implement the method in house, not to consider a method as validated and therefore official. However, the analytical quantification of a toxin and its analogues in a sample is not sufficient in terms of monitoring and requires the toxicity equivalency factor (TEF), since different analogues may have different toxic potencies [48]. This factor compares the toxicity of the analogue to that of the reference compound in the toxin group, so that the concentration of the analogue determined by analytical methods in conjunction with the TEF allows the toxicity contribution to be calculated and expressed as toxin equivalents. For adequate TEF estimation, it is necessary to appropriately define the toxicity of each compound.

For instance, a minimum lethal dose for TTX of 2 mg has been assumed in humans historically [52,53,54], and this value is still in the literature even though a much lower LD_50_ of 232 µg/kg and a NOAEL of 75 µg/kg have been demonstrated after oral administration of a TTX single dose [39]. In addition, potential nephrotoxic and cardiotoxic effects have been observed at a TTX dose of 125 µg/kg after repeated oral administration [45]. Currently, the European Food Safety Authority (EFSA) has recommended a safe concentration of TTX below 44 μg of TTX equivalents/kg of shellfish meat in fishery products [55]. However, EFSA also highlighted the need to re-evaluate the seafood safety risk to consumers since recently reported results pointed to potential harmful effects of chronic low oral doses of TTX. This requires further and detailed studies, especially considering the possible synergies between different marine biotoxins with the same mechanism of action [43,45,56].

This same case occurs for ciguatoxins, which, in addition to the lack of certified standards, are not yet regulated in Europe in spite of the fact that the dinoflagellates producers of ciguatoxins are found in our coasts today: the presence of CTXs was confirmed in locally sourced fish in Canary Islands or Madeira [57]. As we and others have demonstrated, ciguatoxins elicited negative shifts in the active potential of voltage gated sodium channels [58,59]. Similarly, for palytoxins and the related compounds, there is continuous expansion of the dinoflagellate producers through mild temperate waters [60,61]. Thus, the toxicological data available for marine biotoxins should be re-evaluated considering all these aspects since new information about the mechanisms of action of toxins is known and new methodologies and purified compounds are available. This is especially relevant when the toxicity equivalency factor (TEF) is linked to a regulatory toxin level, and the establishment of TEFs depends on these data [48].

One of the priorities in order to use of analytical methods efficiently for monitoring purposes is to establish the TEFs that will allow knowing the toxicity of a sample [48]. The replacement of the bioassay by analytical methods has several drawbacks [62]. The unknown toxicity of many toxin analogues as compared with the reference compound is the first problem for setting TEFs. A second problem is the need for one standard for each analogue to be quantified and the lack of enough commercial standards, which has led most analysts to quantify several analogues with one single compound, leading to very large errors in quantification of up to 200% [63]. The third problem is that human toxicological information on these toxins is extremely scarce. Therefore, currently, most of the TEFs used by regulatory agencies are derived from in vivo toxicity and by intraperitoneal administration of the compounds in mice due to the lack of enough certified toxins [48]. Thus, accurate TEFs are essential for the control and establishment of regulatory limits for related compounds. It is noteworthy that the scientific opinions of European Food Safety Authority (EFSA) for marine toxins are always generated by an expert group that reviews the existing literature for each toxin group. For example, the revision of TEFs for tetrodotoxin performed by EFSA [55] was based on acute oral toxicity data that was obtained just in time for the opinion [39]; without this study, it would not be possible to set any risk value. This shows the imperative need for toxicological studies to properly regulate the presence of phycotoxins. However, for many years, EFSA has been highlighting a series of shortcomings that affect the legal limits and the monitoring of regulated marine phycotoxins in Europe [64]. Although these toxins limits are still used today, more studies are needed to improve consumer safety. Related to that, PTXs have been removed from the list of marine biotoxins to be analyzed in live bivalve mollusks in EU [65]. This legislative change is a consequence of the absence of reports of adverse effects in humans associated with PTXs [66]. In other words, a toxin should not be regulated based solely on the lethality by mouse bioassay. Caffeine is a clear example of this overstated policy; if caffeine were present in shellfish, it would be regulated as a toxin, since it is lethal by mouse bioassay.

The following needs were identified by EFSA:

1. Establishment of TEFs based on acute oral toxicity data including analogues with toxic relevance at the levels in which they are present in mollusks;
2. Information is needed on genotoxicity, oral toxicity, and toxicity mechanisms for some groups of toxins;
3. Information is needed on the combined toxicity of different groups of toxins that are usually present in mollusks.

During the last decade, different authors have highlighted the limitations associated with the use of the current TEFs implemented by the European legislation to quantify the toxicity of marine phycotoxins present in fishery products [38,67,68,69]. Among the problems associated with the use of current TEFs for marine toxins monitoring are the following:

1. Most of the current TEFs are based on the acute effects of toxin intraperitoneal (i.p.) injection to mice, but these values do not reflect the oral absorption, which is the relevant route for the effects of marine phycotoxins on human health;
2. The majority of TEFs used today have been estimated using toxins of unknown origin and purity, and therefore generate discrepancies, recognized by EFSA [64]. In fact, the need for use of certified reference materials (CRM) for the different toxin analogues in TEFs determination is highlighted by several extensive reviews on marine toxins TEFs [48,70]. This is still a difficult problem to solve due to the absence of toxin CRMs for many analogues in the previously described toxin groups. In recent years, the commercialization of ISO 17034 certified reference materials for some marine toxins has been guaranteed in Europe through commercial channels (www.cifga.com; accessed on 11 January 2022);
3. Current TEFs for marine neurotoxin proposed by EFSA have been obtained using differently purified toxins and quantifying the amount of toxin according to a different criterion in each laboratory, which increases the diversity and disparity of the data collected by EFSA. This fact should be amended at present. In the case of working with purified toxins from mollusk samples, the Standard Operating Procedures (SOPs) of the European marine biotoxin reference laboratory harmonize the extraction of toxins from mollusk samples and the realization of the corresponding analytical or biological determinations.

In summary, despite the fact that, during the last 10 years, several reports have reviewed the limitations for the use of current TEFs for monitoring marine neurotoxins with analytical methods [39,40,48,70], TEFs not obtained through oral administration of the toxin are still being used at present to determine the toxic load of the samples obtained from fishery products. The limitations of current TEFs in providing adequate assessment of marine phycotoxin-related toxicity have recently been collected in a technical report jointly prepared by FAO/WHO [70]. The following points summarize the drawbacks and recommendations reflected in this report for the use of TEFs as indicators of the toxic load of samples contaminated with marine phycotoxins:

1. First, the absence of correlation between the toxicity obtained by MBA and the acute oral toxicity is highlighted [71]. In general, compounds administered i.p. are absorbed quickly and completely from the peritoneal cavity, while the oral administration can decrease the absorption of many substances, and therefore the i.p. route would provide a much higher toxicity than the real one. The opposite occurs when the toxin is metabolized to a more toxic oral analogue: the MBA would give a lower toxicity, for example, in the case of neosaxitoxin that is more toxic than saxitoxin [72] and other toxins from the group of paralytic toxins [73]. In fact, these two studies with paralytic toxins and other studies with diarrheic toxins [39,40] emphasize the need to review actual TEFs using toxin CRMs and the oral route for toxin administration in order to determine reliable TEFs useful to use analytical methods for neurotoxin monitoring [48].
2. In certain cases, TEFs have been established after measuring in vitro toxicity or cellular effects of the toxin [74,75,76]. Although these studies do not take into account neither the absorption nor the metabolism or elimination of the toxins in vivo, these data have also been taken into consideration by EFSA to establish the current TEFs [64] even when there is no approved in vitro model to evaluate the toxicity of paralytic toxins. In fact, the PSP TEFs reported by FAO/WHO are a combination of in vitro effect on human sodium channels with oral toxicity in mice [70].
3. Although oral toxicity is the relevant parameter to establish TEFs for marine toxins, special caution is necessary, since, although the Organization for Economic Cooperation and Development (OECD) guidelines for determining acute toxicity OECD 420-Acute Oral Toxicity-Fixed Dose Procedure and OECD 425- Acute Oral Toxicity-Up-and-Down-Procedure establishes the administration of the chemical compound by gastric tube, the semisolid content of the stomach of the rodents can facilitate that the toxin is absorbed quickly in the duodenum instead of mixing with the stomach food. In the case of marine toxins, it seems more convenient to administer the toxins in the food to facilitate toxin ingestion with the food in a short time. In fact, TEFs obtained by forced feeding (gavage–gastric tube) and voluntary consumption of food [73] may show differences.

## 3. Mechanism of Action and Toxicity: The Need for Predefined Toxicological Criteria

Marine phycotoxins are bioactive compounds streamlined to act fast at very low concentrations. Most of them are highly specific for key physiological targets such as ion channels, enzymes, pumps, or cellular membrane receptors (Figure 2). Toxicity and consequently human poisonings are generally related to the specific interaction of these toxins with their targets. In addition, these characteristics make them ideal candidates for basic research as well as biotechnological applications. It must be emphasized that data research in this field is advancing quickly even though there are still many unknown issues. The mechanism of action of some marine phycotoxins remains elusive. Furthermore, precise relationship of some historically assumed toxin actions and associated toxicity is lacking. Consequently, this review explores the available knowledge on these topics, identifying the gaps and highlighting future challenges and research priorities.

### 3.1. Marine Phycotoxins Acting on Voltage-Gated Sodium Channel

Voltage-gated sodium channels (VGSCs) are essential in the generation and transmission of action potentials in excitable cells such as neurons and muscle cells [77]. VGSCs are formed by a core α-subunit with four repeated domains (I-IV) coupled to β regulatory subunits. In those domains, two highly conserved regions (P1 and P2) partially re-enter cellular membrane from the outer side. In these segments, a ring of four amino acids, Asp–Glu–Lys–Ala (one from each domain), forms the DEKA motif, which comprises the “selectivity filter” allowing Na^+^ influx [78]. There are eight binding sites targeted by many toxins, including several phycotoxin groups [77,79]. Moreover, ten isoforms of VGSCs have been identified in humans (Na_v_1.1–Na_v_1.9 and Na_x_), nine of them functional [77]. Toxins acting on VGSCs are PSTs, TTX, BTXs, and CTXs. BTXs and CTXs activate site 5 of VGSCs, while STX and TTX bind to site 1 and block ion conduction (Figure 2).

**Figure 3:**
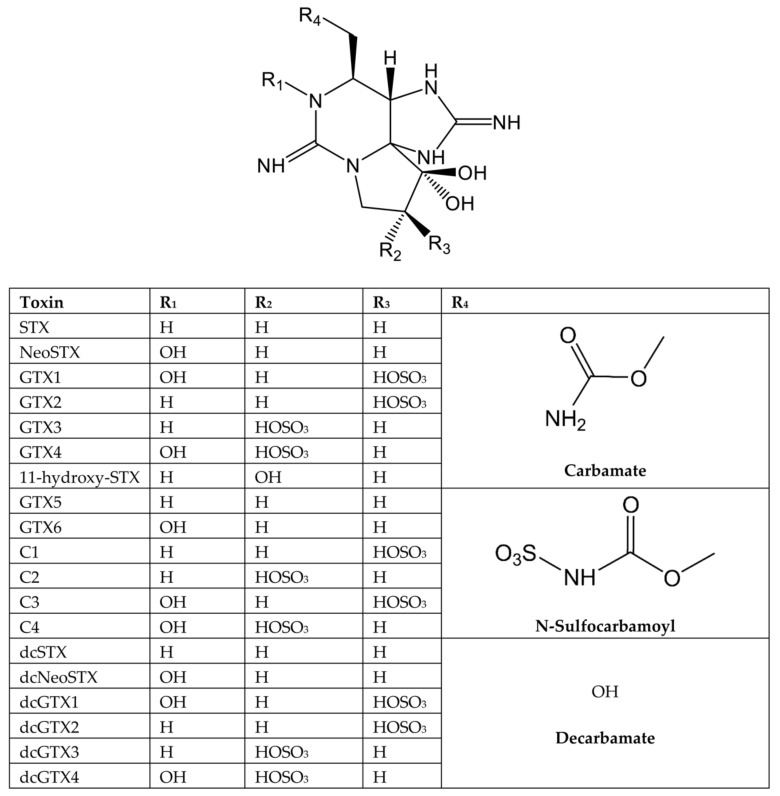
Chemical structure of the main saxitoxin analogues.

The PSTs group of toxins includes more than 50 analogues. Several subgroups can be defined based on their chemical structure; of great importance are carbamate, N-sulfoyl-carbamate, and decarbamoyl derivatives, though other derivatives such as deoxydecarbamoyl have been identified (Figure 3) [5,6]. STX binds to a region placed on the outer side of the channel known as Site 1, blocking Na^+^ entry into the cell (Figure 2) [7,80,81]. Even though STX–VGSC interaction has been known for decades, binding data were originated from mutational cycles, electrophysiological recordings, and prokaryotic or chimeric VGSCs in combination with computational analysis [74,80,82,83]. Only recently has the structure of VGSC Na_v_1.4 been fully elucidated [77,83], followed one year later by high resolution STX-human Na_v_1.7 interaction structure [81]. This study determined the amino acids directly binding to the neurotoxin. STX and TTX bind to VGSCs but with different range of affinities for each isoform [32]. Thus, the sodium channels alpha subunits (Na_v_) Na_v_ 1.1 (EC_50_ 6 nM), Na_v_ 1.2 (EC_50_ 18 nM), Na_v_ 1.3 (EC_50_ 4 nM), Na_v_ 1.4 (EC_50_ 25 nM), Na_v_ 1.6 (EC_50_ 6 nM), and Na_v_ 1.7 (EC_50_ 24.5 nM), are highly TTX-sensitive, while Na_v_ 1.5 (EC_50_ 5.7 µM), Na_v_ 1.8 (EC_50_ 60 µM), and Na_v_ 1.9 (EC_50_ 40 µM) are considered TTX-resistant [84].

Given the considerable number of STX and TTX analogues and the fact that new compounds continue to be added to the list [85], gathering data of binding affinities to VGSCs along with their oral toxicity in vivo entails a major challenge [86]. This further enlarges the resources needed to evaluate potential toxicity of these toxins, which may be reduced by knowledge of VGSC structure in combination with bioinformatic tools, though experimental data are essential to confirm computational predictions. Aside from analogues affinity and toxicity data, a growing concern arises from additive effects of co-occurring toxins [87].

The symptomatology observed in vivo is in accordance with the action of these VGSCs targeted toxins. As a consequence of Na^+^ influx inhibition, neuromuscular complications are features of PSP, which can compromise breathing due to muscle unresponsiveness, leading to paralysis of the diaphragm and resulting in lethality in extremely severe cases [88]. TTX poisoning presents with similar symptoms, consequently to the shared mode of action [8]. Toxicity variability among PSTs analogues may result from affinities to VGSCs isoforms [89,90].

Among the main groups of marine toxins capable of causing effects in humans, PSTs are likely the most dangerous for the severity of symptoms reported in seafood consumers. Bivalve mollusks are the traditional vectors, although PSTs have also been detected in some gastropods, crustaceans, and less frequently in fish [91,92]. Shellfish feeding on PST-producing phytoplankton species can accumulate the toxins, in most cases without exhibiting adverse effects themselves [93]. Additionally, PSTs are heat stable; thus, cooking does not destroy the toxins. After ingestion of bivalves with STX, absorption occurs mainly in the gut, followed by distribution to the remaining organs and tissues over time [94].

PSTs analogues have different toxic potencies [95,96]. This is important since bioconversions of PSTs may occur in phytoplankton, bivalves, and humans [97]. One of the ways to establish the toxicity relationship among analogues is through the TEFs. In the PSP group, this factor compares the toxicity of the analogue to that of STX. The concentration of the analogue determined by analytical methods in conjunction with the TEF allows the toxicity contribution to be calculated and expressed as STX equivalents [96].

According to FAO and WHO, the toxic character of the most well-known PSTs analogues varies as follows: carbamate toxins are highly toxic, including saxitoxin (STX), neosaxitoxin (NeoSTX), and gonyautoxins (GTX1-4). Regarding decarbamoyl analogues (dcSTX, dcNeoSTX, and dcGTX1-4) and deoxydecarbamoyl analogues (doSTX, doGTX2, and doGTX3), their toxicity is intermediary. The least toxic derivatives are the N-sulfocarbamoyl toxins GTX5, GTX6, and C1-C4. Using certified PSTs on neuronal cultures and on mouse bioassay (MBA), a list of TEFs was proposed by EFSA (Table 1) [5].

TEF values were reevaluated by oral administration (gavage or feeding) and, as a result, TEFs for dcSTX and dcNeoSTX were lower when determined by oral toxicity than by MBA. However, oral TEF for NeoSTX was higher compared to the value obtained by the MBA [72]. It is interesting to note that there is a better match between the TEF obtained with in vitro methods using toxin potency on Na_v_ subtype 1.2 channel blockage [74] and with oral toxicity in mice [72] than by MBA (Table 1). Related to that, conversion of dcGTX1&4 within the digestive tract to more toxic congeners may explain their high relative toxicity by feeding compared to that determined intraperitoneally [98,99]. There are also some gaps; for instance, TEFs have not been disclosed for the most recently discovered toxins yet, namely those of the M-series, belonging to both the carbamate group (M2, M4, M6, M8, M10, and M12) and the N-sulfocarbamoyl group (M1, M3, M5, M7, M9, and M11), although the few data collected so far seem to suggest a low toxicity among them.

**Table 1:** Toxicity equivalency factors (TEFs) for PSTs depending on the testing assay.

Toxin	TEF EFSA [5]	TEF FAO [70]	TEFs Based on Oral Gavage	TEFs Based on Voluntary Feeding	TEFs Based on In Vitro IC_50_ for Each Na_v_ Subunit [74]						
					Na_v_1.1	Na_v_1.2	Na_v_1.3	Na_v_1.4	Na_v_1.5	Na_v_1.6	Na_v_1.7
STX	1	1	1 ^a^	1 ^a^	1	1	1	1	1	1	1
NeoSTX	1	2	1.7 ^a^	2.54 ^a^	6.4	2.63	2.85	2.6	38.6	1.2	5
GTX1	1	1									
GTX2	0.4	0.4									
GTX3	0.6	0.6									
GTX4	0.7	0.7									
GTX5	0.1	0.1	0.063 ^b^	0.064 ^b^	0.015	0.014	0.08	0.18	10.7	0.11	4.08
GTX6	0.1	0.05	0.038 ^b^	<0.017 ^b^							
GTX1&4			0.74 ^a^	0.93 ^a^	0.96	0.54	1.6	0.57	14.4	1.4	9.29
GTX2&3			0.53 ^a^	0.57 ^a^	0.2	0.39	0.64	0.32	3.87	0.15	0.27
dcSTX	1	0.5	0.46 ^a^	0.37 ^a^	0.07	0.25	0.08	0.16	0.96	0.96	4.6
dcNeoSTX	0.4	0.2	0.22 ^b^	0.22 ^b^	0.001	0.1	0.024	0.001	0.73	0.25	0.33
dcGTX2	0.2	0.2									
dcGTX3	0.4	0.4									
dcGTX1&4				0.1 ^c^							
dcGTX2&3			0.17 ^b^	0.11 ^b^	0.04	0.05	0.22	0.01	3.3.	0.02	3.1
C1		0.01									
C2	0.1	0.1									
C3		0.01									
C4	0.1	0.1									
C1&2			0.034 ^b^	0.043 ^b^	0.008	0.013	0.25	ND	2.6	0.09	0.1
C3&4			0.028 ^b^	ND ^b^							
11-hydroxy-STX	0.3										

IC_50_: inhibitory concentration 50, ^a^ data from Munday et al. (2013) [72] ^b^ data from Selwood et al., (2017) [73]; ^c^ data from Boundy et al., (2021) [98].

Upon ingestion of STX-bearing shellfish, the severity of PSP symptoms depends on the analogues and the doses ingested [94]. The poisoning effects occur quickly; the primary site of STX action in humans is the peripheral nervous system, causing a fast start of symptoms: numbness or a tingling sensation around the lips and tongue, which appear in less than 1 h and are due to local absorption of the PSP toxins through the buccal mucous membranes. Frequent symptoms are also a stinging sensation in the toes and fingertips, nausea, vomiting, diarrhea, dizziness, and headaches [100]. In severe poisoning, death may occur within 24 h of ingestion [3].

PSTs could also be fatal for marine wildlife. PSTs can enter the food web when toxin-producing dinoflagellates or cyanobacteria are ingested by shellfish, copepods, or other invertebrates and these, in turn, are consumed by larger organisms. Ingestion of PSTs by mammal and bird species can result in muscular weakness, motor incoordination, respiratory paralysis, and death [101,102].

In order to protect human health and to promote the trade of safe seafood, maximum permitted levels of STX in seafood have been established by regulatory authorities in many countries with the recommended regulatory level in CODEX of 800 μg STX equivalents/kg shellfish flesh [49]. The current regulatory limit for PST is based on the acute reference dose (ARfD) of 0.5 µg STX eq/kg body weight (bw) proposed by the European Food Safety Authority [65,103]. This limit seems appropriate in accordance with studies performed in mice to mimic human feeding behavior and diets containing STX [104]. However, oral toxicity assessment of natural toxin mixtures would reinforce consumer safety. The importance of the toxicological knowledge on PSTs should be highlighted, also considering the potential human chronic exposure [105]. A recent study demonstrated that daily exposure for 3 months to low levels of STX could cause significant cognitive deficits and neuronal cell cutbacks. The alterations of hippocampal sphingolipid metabolism and hippo signaling-pathway-related proteins may be involved in the STX-induced nerve damage [106]. It was verified that STX can cross the placental barrier and reach the fetal brain. This contributes to the understanding on toxic effects of these neurotoxins on the development of animal neuronal cells. Currently, there are no antidotes or therapies for PSP. Mortality induced by PSTs depends on the prompt recognition of PSP symptoms, which prevent complications and patient deaths. Clinical measures are taken to try speed up detoxification; the use of activated charcoal to remove unabsorbed toxins or cleaning gastric contents may be considered. Rapid intervention includes fluid therapy, assisted ventilation, and hemodialysis.

TTXs are also extremely potent toxins with 25 analogues. They induce paralysis of muscles and even death through cardiorespiratory failures. TTX and analogues were recently detected in marine bivalves and gastropods from European waters [107,108] being a serious threat to human health. It should be considered that the toxicity of analogues is lower than TTX as reported based on intraperitoneal toxicity to mice [109]. However, even though acute oral toxicity of TTX was already reported with LD_50_ of 232 µg/kg, to date, no studies evaluating the oral toxicity of TTX analogues have been released.

In the EU TTXs are not monitored; the only relevant requirement in the current legislative framework is that fishery products derived from poisonous fish of the family Tetraodontidae must not be placed on the market [38,110]. EFSA proposed a safe concentration lower than 44 µg TTX eq/kg of shellfish meat [55]. However, TTXs levels detected in shellfish in the EU are often higher than this value, which indicated that seafood is in danger of being contaminated with this hazardous toxin and that appropriate measures are possibly required to protect human health [111]. Therefore, TTXs could be a future concern in Europe, as well as new global health risk due to the spread and prevalence in new geographical regions.

In contrast to PSTs and TTXs, CTXs and BTXs maintain VGSCs in an active form [32]. CTXs and BTXs bind to site 5 of VGSCs, inducing an open state, subsequently allowing Na^+^ passage inside cells. VGSC site 5 is comprised of the transmembrane segments S6 and S5 of the α-subunit domains I and IV, respectively [32,112]. CTXs bind to this site from the intracellular side of VGSCs (Figure 2) [77]. Even though the region binding CTXs is known, the detailed interaction and conformation of CTXs–VGSCs binding structure has not been elucidated [77]. Similarly to other toxins, the activation of VGSCs by CTXs and BTXs induces action potential repeated firing and ion imbalance. It would be of interest to unveil CTX binding structure to VGSCs and variations with different analogue structures. In addition to their activation of sodium channels, CTXs have been shown to partially inhibit voltage-gated potassium channels (K_v_), thus further increasing the membrane excitability [113]. Therefore, and secondary to sodium channel activation, CTXs trigger several cellular effects including swelling, neurosecretion, an increase in intracellular calcium levels and the modulation of gene expression [114].

**Figure 4:**
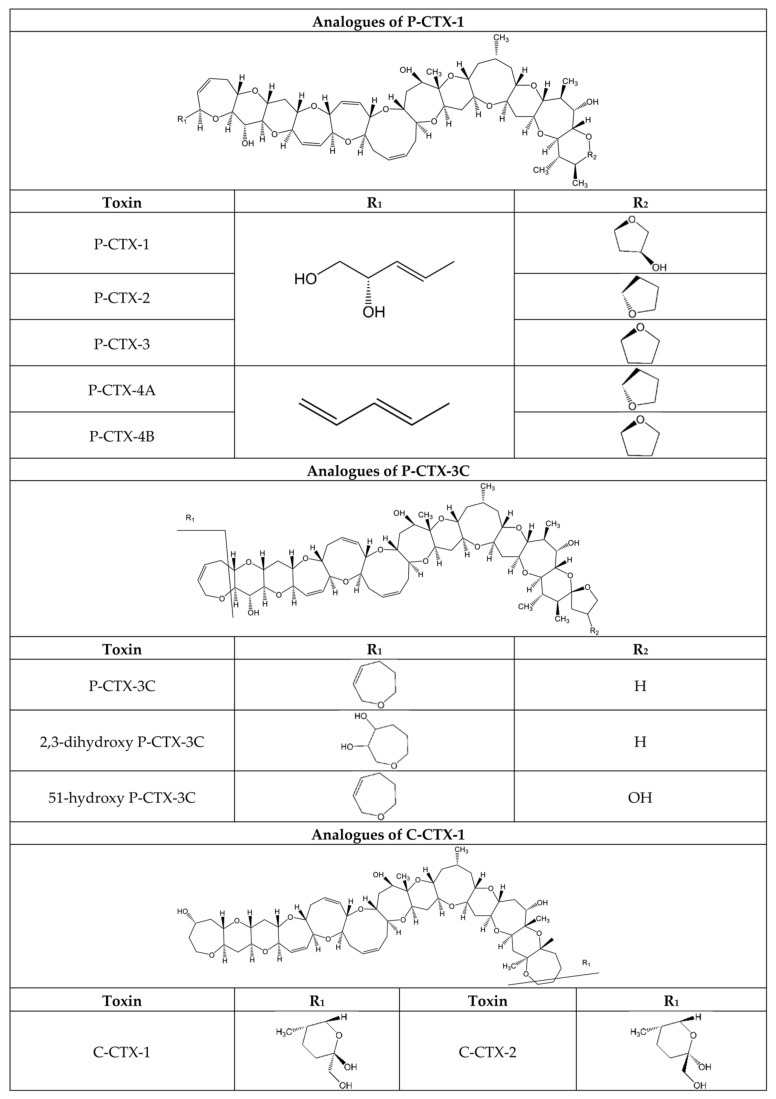
Chemical structure of ciguatoxin analogues.

Some CTXs are produced by *Gambierdiscus* and *Fukuyoa* dinoflagellates [115], but the biotransformation of ciguatoxins in invertebrates and fish has contributed to the more than 30 analogues reported-to date [116,117]. The molecular structures of ciguatoxins found within fish vary with location and historically a prefix is added to the name to distinguish them: P for compounds from the Pacific (e.g., P-CTX-1B) and C for compounds from the Caribbean (e.g., C-CTX-1) (Figure 4) [30]. These structural differences result in Pacific-ciguatoxin-1 (P-CTX-1) being more potent than Caribbean-ciguatoxin-1 (C-CTX-1) [118]. It is worth mentioning that other groups of toxins can co-occur since they are produced by the same dinoflagellate species. These are maitotoxins and compounds such as gambierol [30]. Their mode of action is different from that of CTXs, though new reports shed some light on the effects at molecular level. Briefly, gambierol is a potent blocker of voltage-gated potassium channels (K_v_), both in human T lymphocytes and mouse fibroblasts at nanomolar concentrations [119,120]. It also shows almost full inhibition (>97%) of the potassium channel subtypes 1.2, 1.3, and 1.4 at concentrations between 1 and 1.5 µM [121]. On the other hand, the activation of voltage-gated Ca^2+^ channels (Ca_v_) and the consequent entry of external Ca^2+^ induced by MTX turns it into an important tool for studies in all cellular and physiological processes in which these channels are involved [122,123].

In a recent study, the effects of these toxins in human VGSCs have been analyzed, and gambierol, gambierone, and maitotoxin 3 (MTX3) had no effect on the size of the sodium currents. However, gambierone shifted the activation of VGSCs in the negative direction. The negative shift in the activation also allowed quantifying the low potency of MTX3 [59]. Therefore, gaining knowledge regarding the molecular target of these toxins and its relationship with in vivo toxicity should be addressed.

The relative potencies of CTXs analogues have so far been determined by the mouse bioassay (MBA). However, even using a bioassay, there are many variables that can affect the data from different laboratories since it is a non-standarized method. In addition, these toxicity experiments were carried out with toxins that were not standards. Until better information is available, the panel on contaminants in the food chain adopted the TEFs that appear in the first column of Table 2 [124].

**Figure 5:**
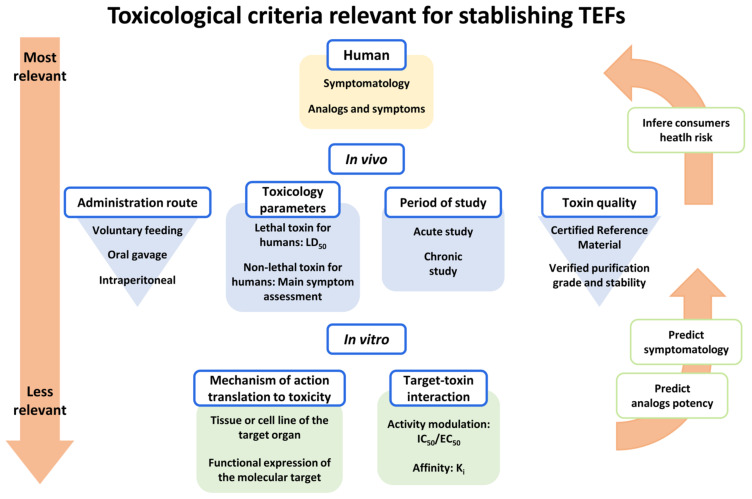
Criteria in which toxicity equivalency factors (TEFs) are established are ranked based on their importance. Firstly, epidemiological data and clinical course reported from poisoning outbreaks along with the identified responsible toxin. Data obtained from in vivo assessments are then considered. Approaches such as the route of toxin administration or toxin quality are taken into account to evaluate relative potencies. Moreover, the toxicological measurement allowing for comparison between analogues should be selected based on symptoms observed in humans. This is, when the toxin causes death, a median lethal dose is recommended; however, the main symptom should be evaluated if no reported cases of fatalities are known. Finally, in vitro experimental reports allow to study the molecular target. The biological system should be corresponding to the observed affected/targeted tissues or organs *in vivo.* Even though the preferential order has been described, our proposal is to consider in vitro and in vivo information complementary for the establishment of TEFs. In vitro assays can also provide essential data that help in understanding the mechanism of action. This implies the relative potency of analogues or the clinical course expected to be observed in vivo. Similarly, in vivo studies are fundamental in determining TEFs even when human poisoning data are available. Modified from FAO/WHO (2016) [70].

The best option is to set TEFs based on human data, but epidemiological information is scarce, and other options should be considered (Figure 5). The lack of biomarkers to confirm Ciguatera diagnosis in humans and the failure by health professionals to achieve proper differential diagnosis in patients clearly explains the scarcity of epidemiological data. Since human exposure is in most cases through toxin ingestion, toxicity data through oral administration to animals are relevant. Recently, the Expert Group on Ciguatera concluded that, due to limited data from oral in vivo studies, it has not been possible to derive TEFs [30]. As was mentioned above, the in vitro toxic potency of these compounds in humans VGSCs could be a good indicator of their toxic effect, and these in vitro data should be also considered for determination of TEFs (Figure 5), although these bioassays have not yet been sufficiently validated for use in risk assessment [30].

CFP is the most prevalent biotoxin-related seafood poisoning. The transfer of CTXs, within and among food webs, is due to their lipid-soluble bio-accumulative properties [28]. Toxic dinoflagellates adhere to algae, coral, and seaweed, where herbivorous fish eat them. Ciguatoxins are transferred through the food web from herbivorous reef fish to larger carnivorous finfish and bioaccumulated as they move up the food chain until they reach humans. The highest levels of toxins are observed in long-lived fish-eating predators [125]. Therefore, slightly higher toxicity in upper trophic level fish suggested biomagnification up the food chain [126]. Many fish species are regarded as potential vectors of CTXs, including but not limited to barracuda, grouper, snapper, amberjack, trevally, wrasse, mackerel, tang, moray eels, and parrotfish [28].

Ciguatoxin is tasteless, odorless, and heat-resistant, so boiling, cooking, frying, freezing, or baking cannot detoxify ciguatoxin-laden fish [127]. Additionally, it is not possible to distinguish a toxic fish from a non-toxic one by appearance, texture, smell, or taste [28]. CTXs are concentrated in the fish head, liver, intestines, roe, and other viscera. Toxicokinetic data indicate that CTXs are readily absorbed and largely distributed to the body tissues, including the muscles, liver, and brain, likely due to their lipophilic nature [30]. It was suggested that the quasi-irreversible binding of CTXs to VGSCs and a potential release from binding sites (tissue or plasma proteins or lipoproteins), may contribute to the persistence and reoccurrence of Ciguatera sensory disorders.

When humans consume fish containing CTXs in sufficient amounts, the expected gastrointestinal, cardiovascular, and neurological symptoms of Ciguatera are classically presented within 1–6 h of fish ingestion [26,32]. The neurological symptoms include paresthesia, dysesthesia, vertigo, and sensory abnormalities such as metallic taste, pruritus, arthralgia, myalgia, dental pain, and cold allodynia, a pathognomonic Ciguatera symptom that is characterized by burning pain in response to a cold stimulus [128]. All CTXs analogues contribute to the neuronal firing, but under some conditions, external sensory stimuli might trigger a Ciguatera crisis, and a negative shift in the activation voltage of the sodium channels could be behind them [59]. Therefore, even though it is well-known that CTXs bind to site 5 of VGSC, the action related to the main clinical symptoms of Ciguatera in humans is not well-defined. It is a challenge to determine if it is related to the sensitization of the sodium channel or lowering of the trigger threshold to a nociceptive stimulus.

The predominance of sensory disorders suggests that CTXs particularly target somatosensory nerves/neurons. The time course of sensory disturbances (i.e., perioral paresthesia, abdominal pain, and then pruritus and pain affecting the whole body including the face) suggests an initial impact on trigeminal and enteric sensory afferents, then dorsal root ganglion (DRG) and trigeminal sensory nerves [114]. This is compatible with absorption through the mouth and intestine mucosa followed by distribution to the DRG and trigeminal neurons. Cell soma in the peripheral nervous system are located in ganglia and are not protected by blood–brain or blood–spinal cord barriers. These toxicokinetic factors could contribute to the preferential sensory toxicity of CTX [129]. Additionally, a variety of gastrointestinal symptoms including abdominal pain, nausea and vomiting, and cardiovascular symptoms, such as heart rhythm disturbances, may also affect poisoned patients. In addition, breastfeeding mothers have reported diarrhea and facial rashes in their infants. This supports the theory that Ciguatera toxins are secreted into breast milk. The toxicity of Ciguatera is generally self-limiting, with gastrointestinal and cardiovascular manifestations only lasting a few days. Some symptoms, mainly neurological, can last days to weeks or even months to years, and, in extreme cases of severity, Ciguatera may cause the death of patients [28,130]. Chronic low-dose exposure to CTXs in humans over time may represent a potential long-term human health risk, as CTXs can bioaccumulate, cause DNA damage, and cross the blood–brain barrier [131,132].

The effects of CTXs on marine fauna are less documented. Ciguatoxins found in the brain, liver, and muscles of marine mammals suggest that they may also suffer from CTX exposure and that these compounds persist within the complex marine food webs [133]. The fish resistance mechanism to CTX is still unknown.

Ciguatera fish poisoning or Ciguatera is mainly encountered in tropical and subtropical areas. Ciguatera also can represent a major source of concern to the tourism industry in endemic regions [28]. However, with the increase in fish imports and tourism, clinical Ciguatera can be found in non-endemic areas [134]. In the recent past, a geographical expansion of CTXs to more temperate areas has been evidenced by factors such as climate change, some anthropogenic activities, as well as the migration patterns of ciguateric fish [135]. Ciguatera is an emerging hazard in European waters (Canary and Madeira islands and the Mediterranean Sea), thus necessitating the adoption of official policies to manage the potential risks [136]. Until now, in the European Union’s fisheries and aquaculture products market, fish with CTX-group toxins are forbidden [124]. In the United States, the current Food and Drug Administration (FDA) guideline for Ciguatera is now listed as 0.01 ng/g for Pacific ciguatoxin and 0.1 ng/g for Caribbean ciguatoxin [30]. Therefore, to adhere to the guidance, the CTX fish content should not exceed these recommended levels. Overall, the effective management of Ciguatera patients is significantly hampered by the lack of a specific antidote, and medical management of acute and chronic Ciguatera in affected patients relies mainly on symptomatic support and diet recommendations [26]. The autonomic dysfunction-based disorders, including digestive and cardiovascular symptoms, resolve spontaneously or are treated effectively. Treatment to relieve the persistent sensory disturbances is lacking. The benefit of mannitol is controversial in Ciguatera poisoning; some clinical trials found no difference between mannitol and normal saline, while other trials have demonstrated improvement of neurologic symptoms after administrating mannitol. The most frequently given advice is not to consume fish weighing more than 2 kg and not eating fish parts such as the viscera, brain, and gonads, where ciguatoxins are mostly accumulated [137].

Regarding MTXs, their implication in Ciguatera is unlikely [30]. Six MTX analogues have been identified: maitotoxin-1 (MTX1), maitotoxin-2 (MTX2), maitotoxin-3 (44-methyl gambierone), maitotoxin-4 (MTX4), desulfo-MTX1, and didehydro-demethyl-desulfo-MTX1. MTXs have been historically considered one of the most toxic marine biotoxins that induced activation of voltage-gated Ca^2+^ channels (Ca_v_) and the consequent entry of external Ca^2+^ [138]. However, recent reports indicated that MTXs have almost no activity on VGSCs [59]. All MTXs are characterized structurally by having at least one sulfate group, giving them increased polarity compared to CTXs. Their higher polarity limits their absorption when ingested [139]. Therefore, although its intraperitoneal administration in mice seem toxic, MTXs’ oral toxicity is almost nondetectable [140], leading to them not be considered compounds responsible for Ciguatera. In addition, their accumulation along the food web is low, and they have not been found in tissue of fish involved in Ciguatera cases [141].

**Figure 9:**
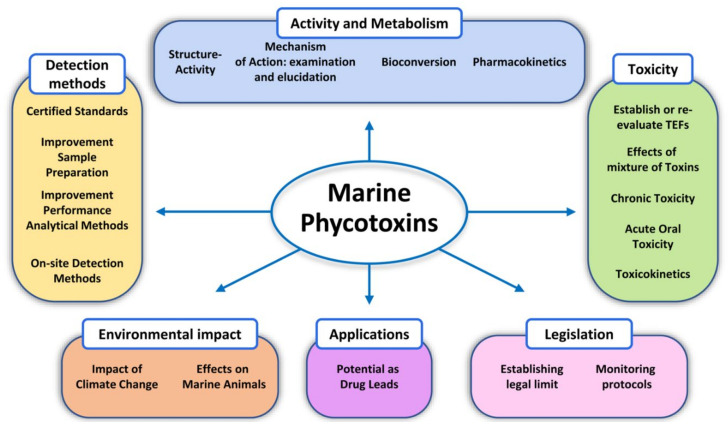
New challenges associated with marine phycotoxins outlined in this review.

Future challenges related with marine phycotoxins acting on voltage-gated sodium channel (sumarised in Figure 9):

- Epidemiology studies;
- Studies of structure–activity relationship of toxins;
- Common criteria in the naming of toxins;
- Reevaluation of preestablished toxicity concepts based on false premises;
- Review mechanism of action responsible for the toxicity of compounds including mechanisms involved in the disturbances that can persist or reoccur many months or even years afterwards;
- Harmonization of criteria to set toxicity parameters to establish accurate TEF values especially of those toxin analogues commonly found in seafood or at relatively high levels;
- Research to better understand the toxins produced by bioconversion in the organisms and their toxicity;
- Information about pharmacokinetics of toxins;
- Toxicity studies with special focus on oral toxicity and on toxin mixtures;
- Studies related to chronic exposure of toxins;
- Information on the occurrence and factors conducive to the accumulation of toxins in marine organisms;
- Common legislative criteria: toxin regulation, implementation of effective toxin monitoring, and management programs for toxins;
- Climate change and its consequences;
- Evaluation of the therapeutic potential of these toxins based on the reversible interaction with the sodium channels.

### 3.2. Marine Phycotoxins Acting on Glutamate Receptors: Domoic Acid and Analogues

Glutamate is the major excitatory neurotransmitter in the central nervous system (CNS), though it is also expressed in peripheral tissues [142]. It binds to ionotropic glutamate receptors (iGluRs) [143], which are also the target for a variety of natural occurring toxins, such as domoic acid (DA) [144]. DA causes amnesic shellfish poisoning (ASP), named after the memory loss observed [145]. DA is a hydrophilic amino acid with several isomers, isodomoic acid A-H and epi-domoic acid [146], but only a few were detected in seafood products [147]. Domoic acid is a known agonist of iGluRs, which maintains them at an open state, triggering neuron excitability (Figure 2) [144]. Three functional classes of iGluRs are currently identified: kainate receptors, AMPA receptors, and NMDA receptors [148]. They are formed by two pairs of dimers constituting a tetrameric structure conformed in a circular manner [149]. Each subunit has three transmembrane regions (M1, M3, and M4) with a partial re-entering loop from the cytoplasm to the membrane. Two large extracellular domains are also defined, i.e., the N-terminal domain and ligand-binding domain (LBD) [149,150]. The structure of DA binding to rat kainate receptors GluK1 (former GluR5) LBD and GluK2 (former GluR6) LBD has been determined at a high resolution [148,151,152]. LBD has a clamshell form to which DA binds between the two “shells” (lobes). Afterwards, it partially closes, leading to channel opening and calcium entrance [149,150]. However, these structures are based on LBD soluble form; thus, these conformation modifications are to be confirmed by elucidating DA structure bound to full length receptors. Additionally, DA is an agonist not only of kainate receptors but also AMPA receptors [144,146]. High-resolution conformational changes induced by DA in AMPA receptors in the LBD or in the full-length receptor have not been revealed yet.

Kainate and AMPA receptors excitability implies Ca^2+^ and Na^+^ influx into neurons, which, in turn, activates NMDA receptors and glutamatergic signaling [144,147]. This is the mechanism through which DA causes ASP symptomatology [146]. Current challenges regarding the DA mode of action rely on structure–activity knowledge and detailed research into toxicology information on peripheral sites. iGluRs activity is complex due to different subunits combinations, desensitization, and auxiliary subunits assembly [143]. Structural modifications induced by DA, not only in the LBD, but also in the whole receptor, will help in understanding the structure–activity relationship. To this regard, data about DA isomers are also missing.

Not all bivalve species have the same capability to accumulate DA; the differences observed can be related to the depuration rate. Most bivalves depurate DA very fast, except the king scallop *Pecten maximus* and the razor clam *Siliqua patula*, which accumulate high concentrations of DA [153]. ASP symptoms usually appear in humans 24–48 h after the consumption of DA-bearing bivalve mollusks. The clinical course is in accordance with glutamate being the major excitatory neurotransmitter in the CNS and its role in the autonomous nervous system as well as the broad distribution of iGluRs in the body [142]. Gastrointestinal signs are usually the earliest onset symptoms and the most frequent ones comprising nausea, vomiting, and diarrhea, among others [154,155,156]. In more severe cases, neurological complications, such as disorientation, confusion, headache, seizures, and memory loss develop. Other peripheral alterations can also manifest, such as cardiac arrythmias, blood pressure instability, or bronchial secretion [154,156]. In addition, difficulty in breathing, coma, and even certain cases of death have also been reported.

DA is a water-soluble small molecule with low transcellular permeability [157]. Most isomers seem to be less toxic than DA [158]. Toxicokinetic data of DA are scarce, but due to its physicochemical characteristics, it is not expected to distribute widely in the body. In laboratory animals, DA following oral dose was absorbed slowly in the gut, limiting its oral bioavailability, and is mainly eliminated unchanged in the urine through glomerular filtration [159]. Neurotoxicity is the critical toxicological effect identified in experimental animals as well as in humans. Toxicity and effects of DA and isomers in the CNS have been evaluated in depth. Following acute DA exposure, laboratory models exhibit progressive symptoms with effects that include activity level changes, gastrointestinal distress, stereotypic behaviors, seizures, and death [160]. Due to its prominent role in ASP, memory has been the focus of most DA research. Both DA doses that trigger most ASP symptoms and asymptomatic DA doses cause adverse learning and memory outcomes, which were reversible in asymptomatic rodents [161]. The high doses of DA damage neurons by over-activating kainate receptors, leading to uncontrolled calcium influxes, and induce cell degeneration in certain regions of the brain, most recognizably in the hippocampus (the memory center of the brain) [162]. In mammals, prenatal and neonatal DA exposure has been linked to abnormalities in electrophysiology and a reduced threshold for chemically induced seizures [163]. It should be noted that research has been focused on the nervous system, leaving behind the understanding of DA effects on peripheral tissues such as cardiovascular, gastrointestinal, and renal impairment [86].

EFSA established an acute reference dose (ARfD) based on human data of acute toxicity from an outbreak of DA poisoning in Canada in 1987, comprising 107 cases [155]. The CONTAM Panel used the lowest observed adverse effect level (LOAEL) of 0.9 mg/kg bw, applying an uncertainty factor of 30 to derive an acute reference dose (ARfD) of 30 μg/kg bw. Because only DA and its diastereoisomer epi-DA have toxicological relevance, the ARfD applies to the sum of DA and epi-DA [146]. Consequently, a TEF of 1 is applicable. Genotoxicity data on DA were inconclusive, but chronic exposure to this toxin seems to have health consequences, making its close monitoring even more important [161]. Regulations developed in the late 1980s, and effective seafood monitoring programs for detection of DA in shellfish implemented by many regulatory agencies worldwide have prevented acute human DA poisonings [18]. The EU legislation sets the regulatory limit for DA in shellfish: 20 mg DA/kg of meat [49]. Shellfish harvesting is closed when monitoring programs indicate DA concentrations over the regulatory limit, leading to direct and indirect economic problems for fisheries and aquaculture. There is no antidote available for ASP, and treatment is supportive. Severe complications from DA intoxication have been especially reported for elderly patients.

Humans have been protected from acute ASP by DA regulatory limits in shellfish, but multiple DA toxicity events have occurred in naturally exposed marine mammals over the past three decades and have caused substantial mortality events [164]. In fact, *Pseudo-nitzschia* blooms could be increasing due to climate change as well as the impact of DA on marine animals [165,166]. Marine mammal exposures are similar to the human oral exposure route, and the symptoms of acute sea lions toxicosis syndrome are analogous to ASP; therefore, sea lions have been invaluable sentinel species in DA research [167].

Future research efforts should aim to further explore the challenging topics (included in Figure 9):

- Studies on the oral toxicity of DA isomers present in seafood.
- The health impacts associated with chronic, low-dose exposure to this prevalent neurotoxin. Results from these studies will also help reveal the human subpopulations with pre-existing conditions who may be more vulnerable to the toxic effects of this compound.
- Studies to further elucidate the toxicokinetic of DA and the role of drug transporters
- Research into DA effects other than neurotoxic (cardiac, renal, and gastrointestinal) especially considering chronic exposure.
- Research in humans and animal models should include studies during pregnancy and in exposed offspring to characterize the relationship between the increasing body burden of DA and related neurodevelopmental effects.

### 3.3. Lipophilic Marine Phycotoxins

Lipophilic marine phycotoxins are natural metabolites produced by dinoflagellates, which can be extracted from bivalve tissues using organic solvents. Structurally, they belong to five different groups: okadaic acid, including okadaic acid (OA), and dinophysistoxins (DTXs); azaspiracids (AZAs); pectenotoxins (PTXs); yessotoxins (YTXs); and cyclic imines (CIs).

**Figure 6:**
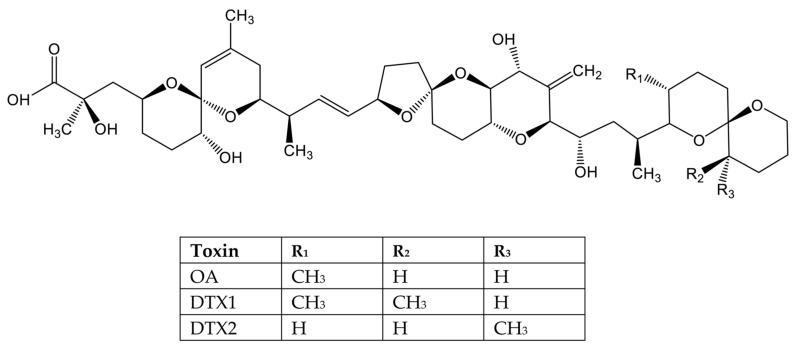
Chemical structure of the main okadaic acid analogues.

The OA group comprises cyclic polyether fatty acids including DTX1 and 2 (Figure 6), as well as their esterification products, referred to as DTX3. They are inhibitors of serine/threonine protein phosphatases (PPs) such as PP2A and PP1 [168] among others (Figure 2), resulting in the hyperphosphorylation of many cell proteins, which, in turn, leads to effects on several pathways [169,170]. The structural conformation of OA-PP1 binding has been resolved by crystallography at a high resolution [171]. These phycotoxins inhibit PP2A preferably to PP1; hence, conformation binding to PP2A was later elucidated [172]. The structure binding conformation was also studied for DTX1 and DTX2 [173]. These toxins bind to a hydrophobic groove close to the active site of PP2A catalytic subunit [171,172,173]. A two amino acid variation in this region leading to loosen ends of the pocket in PP1 would explain the increased affinity for PP2A over PP1 [172]. As mentioned above, OA and DTXs interfere with other PPs such as PP5 or PP6; however, the binding conformation of OA with either PP5 or PP6 has not been reported.

Despite proteins’ dephosphorylation by PPs being essential in modulating the activity of a wide variety of enzymes and tissues [174], their relationship with gastrointestinal dysfunction is not clear. Other PPs inhibitors do not elicit similar effects to OA toxins in vivo [99]. As the most prevalent symptom, diarrhea can be the result of complex mechanisms, and different activation pathways can be implicated [175,176]. Results from in vitro studies had suggested that the potent pro-absorptive peptide neuropeptide Y (NPY) was altered after OA treatment in a neuroblastoma cell line [177]. However, NPY administration prior to OA did not modify OA-induced poisoning in mice, but in the same study, serotonin was directly involved in OA-induced diarrhea [178]. The secretory role of serotonin in the pathophysiology of diarrhea has been largely reported [176,179]. On the other hand, an in vitro study in a model of intestinal barrier shows a protective role of enteric glial cells with regards to OA altered permeability [180]. Enteric glial cells have been related to secretory outcomes in the intestine [179], and whether their activation plays a role in OA diarrheagenicity is to be studied in vivo. Therefore, OA could act by modifying the crosstalk between the enteric nervous system and the intestinal epithelial cells for the regulation of homeostasis, gut functions, and intestinal barrier permeability through changes in the release of various mediators. Based on these recent reports, the need to review the mechanisms of DSTs toxicity should be emphasized.

Diarrhetic shellfish poisoning (DSP) is a gastrointestinal disease associated with the ingestion of filter-feeding shellfish that have ingested OA producer dinoflagellates [13], although other shellfish, such as crabs, can also become toxic. The structural integrity of DSTs remains intact after cooking [181], and their presence in shellfish flesh does not appear to alter the organoleptic profile [182]. DSP includes incapacitating diarrhea, nausea, vomiting, abdominal pain, and, in some cases, chills and fever lasting 3 days on average, but it is not lethal [13,14]. However, the impact DSPs have across marine food webs, including commercial finfish and shellfish, is poorly defined [183].

Despite the efforts and deep research into DSTs toxicity at different levels, the molecular mechanism of action responsible of poisoning was not well-defined [184]. OMIC techniques may be a valuable tool in understanding OA-altered pathways [184]. After ingestion of DSTs-bearing bivalves, toxins are localized mainly at the gastrointestinal tissues [178]. They cause diarrhea stimulating Na^+^ secretion by intestinal cells, leading to intraluminal gastrointestinal (GI) fluid accumulation and abdominal cramping [14]. OA has been shown to induce intestinal toxicity in mice with cell detachment, fluid accumulation, villous atrophy, inflammation, and dilatation of the intestinal tract [185]. Cytotoxicity of OA is mainly manifested as change in cell morphology, destruction of the cytoskeleton, variations in the cell cycle, and induction of apoptosis [186,187,188]. These alterations have been traditionally related to OA inhibition of PPs activity [189,190]. However, the broad symptomatology of DSP cannot be attributed only to inhibition of PPs [72,178,180]. Due to intestine complex structure and tight regulated physiology, understanding the interplay between different components (e.g., enterocytes and the enteric nervous system) in OA response might shed some light to OA mode of action. Beyond the GI tract, other organs can be affected by okadaic acid [72]. Exposure to OA leads to reorganization of cytoskeletal architecture, loss of intercellular communication, and apoptosis in liver [191,192], while a study in rats showed no acute cardiotoxic effects of OA and DTX1 [193]. A variety of OA toxic effects, including genotoxicity, angiotoxicity, immunotoxicity, and embryotoxicity, have been also reported [14,188,194]. Interestingly, the nervous system is sensitive to OA although it is not classified as a neurotoxin. It has been reported that OA can cause neuronal cell death by inducing hyperphosphorylation of a variety of microtubule-binding proteins, especially Tau protein and neurofibrillary tangles formation, resulting in changes in the neuronal cytoskeleton in vitro and in vivo [195]. It has been also demonstrated that OA can produce spatial memory impairment and neurodegeneration and cause hippocampal cell loss in rats [196].

Not only is the OA molecular target of interest, but chronic exposure assessment is also demanded [184]. The chronic toxic potential of DSTs is less understood, although long-term exposure to OA is linked to increased risk of cancer [197]. There are studies that correlate shellfish consumption with the incidence of gastrointestinal cancer in the Spanish and French population of the coast [198]. This possible association agrees with the alterations in the pattern of expression of 10 genes related to carcinogenic processes in SH-SY5Y neuronal cells exposed to OA [182]. Poisoning with toxins from the OA group at sub-regulatory levels could have long-term adverse effects on the digestive tract in people, leading to an increased risk of bacteriosis, likely from an existing resident gut symbiont or pathobiont [199,200]. The disruption of epithelial integrity by OA may affect the colonic microbiota, which, in turn, leads to various diseases such as colorectal cancer [201]. In addition, further research is needed in the toxicologic evaluation of toxins mixtures on behalf of consumers safety [87,184]. DSP treatment is supportive as victims recover without any special therapy after several days. Until now, no sequelae have been reported.

In general, OA, DTX1, and DTX2 are produced by the microalgae, while DTX3 is present only in shellfish [202]. The metabolism of OA/DTXs in shellfish leads to extensive conversion to derivates DTX3 (7-O-acyl fatty acid esters and okadaates), which, although appearing to be of somewhat reduced toxicity, are believed to be largely converted back to the free toxins during digestion. This DSTs conversion, leading to multitude of compounds potentially present in shellfish, complicates the determination of overall toxicity. It should be noted that the main DSTs analogues differ in toxicity, as indicated in several studies performed in vitro with a range of cell lines [203] as well as in vivo in rodents [40,204]. TEFs are also used to determine the concentration of DSTs in shellfish, converting the amounts of individual toxins calculated by analytical methods to OA equivalents [62,65]. TEFs proposed by EFSA are derived from the PP inhibition potency and lethal intraperitoneal doses in mice (Table 3) [103]. However, PP binding affinity is not the only factor important for determining the relative toxicity of OA analogues to human consumers. All OA actions contribute to the final toxicity observed in vivo. Recent studies indicate that analogues’ oral toxicity is DTX1 > OA > DTX2 with TEF values based on oral lethal toxicity: OA = 1, DTX1 = 1.5, and DTX2 = 0.3 [40]. Therefore, because human exposure to OA occurs by ingestion, the current TEF should be reevaluated for regulatory purposes to properly estimate OA equivalents in edible shellfish [70].

To minimize the potential health risk for consumers, several measures have been implemented in many countries including the regular monitoring of shellfish, the establishment of regulatory limits for some lipophilic marine phycotoxins in seafood, and temporary bans on shellfish harvesting whenever toxins exceed the safety limits [207]. The European Union has defined a regulatory threshold that allows a maximum contamination of 160 μg OA eq/kg shellfish flesh. DTX3 are considered in the regulatory framework by including a base-hydrolysis step during sample preparation for toxin detection [170]. Risk of acute intoxication with the current legislation and monitoring system is very low since they protect the population from DSTs acute effects [207]. However, the high persistence of the phytoplankton populations that produce this kind of toxin in many geographic areas [208] indicates that many shellfish consumers may be regularly exposed to low levels of DSTs. This highlights the importance of understanding the health impacts associated with chronic exposure to sub-regulatory levels of DSTs.

**Figure 7:**
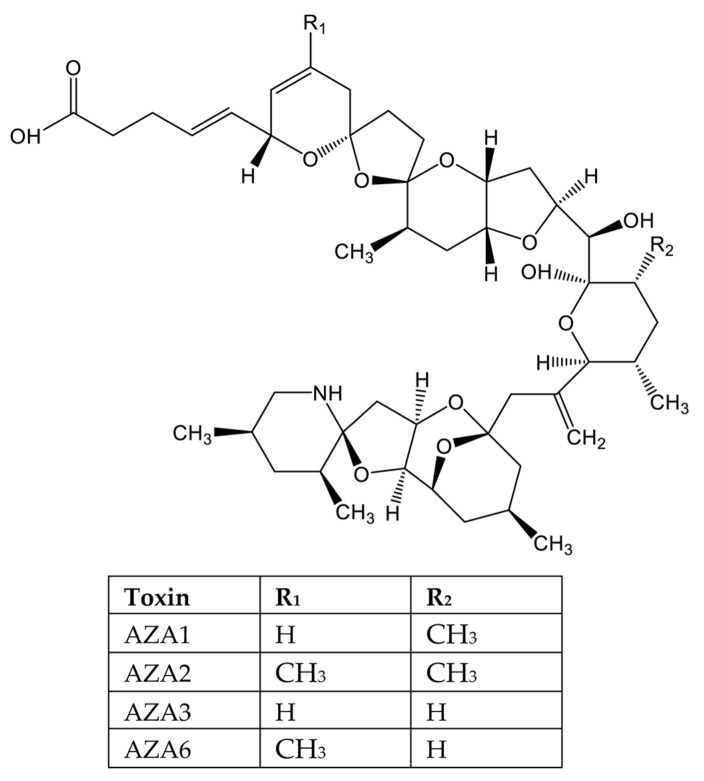
Chemical structure of azaspiracid analogues.

Azaspiracids (AZAs) are a group of polyether lipophilic biotoxins gathering more than 40 analogues, from which AZA1 is the reference compound [19,209]. Hitherto, efforts were focused on analogues AZA1, AZA2, and AZA3, with limited data regarding the remaining derivatives (Figure 7) [86]. Even if the structure of some analogues has been elucidated, the mechanism of action remains elusive (Figure 2). A variety of studies have reported alteration in several pathways under AZAs treatment both in vitro and in vivo. Some of the features determined in vitro comprise cytoskeletal reorganization, apoptosis induction, and mitochondrial and nuclear impairment [16,210,211], depending on dose, experimental time, and cell line. Interestingly, AZAs effects on ion channels have also been described. In hepatocytes, mitochondrial dehydrogenases activity is enhanced by AZAs1-3. Research pointed that these phycotoxins at micromolar concentrations decreased potassium currents acting as open state blockers of *ether-à-go-go* potassium channel (hERG) [212]. In the same line, three VGSCs isoforms are partially blocked in vitro by AZA1, AZA2, and AZA3 [211]. A recent publication reported the interaction of azaspiracids with volume-regulated anion channels (VRAC). Chloride currents’ amplitude is not only increased under AZAs treatment but also significantly diminished when cells are exposed to a selective VRAC inhibitor [211]. Several of these reports might provide some insight to in vivo toxicology. For instance, AZAs modifying hERG activity in vitro could explain at least in part the cardiotoxicity observed in rats after intraperitoneal treatment [213,214,215]. Another example could be hepatocytes’ K^+^ and Cl^-^ channels modification in vitro with liver affection in mice following oral exposure to these biotoxins [215,216]. Cytoskeletal rearrangement in the intestinal cell model could also be related to intestinal fluid accumulation in mice [16,215].

Concerning AZAs, all encouraging in vitro effects should be confirmed in vivo in order to unveil their molecular target. In spite of all data, translation to human symptomatology is still a major challenge. Human poisonings that were ascribed to AZAs are currently limited to the ingestion of azaspiracid-laden mussels. AZP typically comprise gastrointestinal alterations: nausea, vomiting, diarrhea, stomach cramps, and even headache, but no deaths have been reported [217]. The lipophilic properties of AZAs ensure broad capabilities to cross cell membranes and interact with many biological structures. Several studies have underlined the complexity of AZAs effects, since they induce different responses depending on the experimental models. In vitro toxic effects of AZAs at the organ, cellular, and molecular levels revealed the inhibition of neuronal bioelectric activity, alteration in cell–cell adhesion, generation of autophagosomes, ATP depletion, upregulation of proteins involved in energy metabolism, and Golgi apparatus disruption [16,218,219].

It should be also considered that AZAs accumulate in shellfish tissues and have the potential to be metabolized similarly to other lipophilic toxins [220]. However, AZA analogues have different toxicity, probably due to their specific molecular structures [221]. To characterize in vivo toxicity, research has been performed with AZAs after oral, intravenous (iv), and i.p. administration. Most studies are limited to AZA1 with lethality at doses ranging from 250 to 775 µg/kg [222,223]. Recently, a comparative acute oral toxicity study on AZA1, −2, and −3 was performed in mice [215]. The lethal potency was AZA1 > AZA2 > AZA3, and the TEFs derived from LD_50_ were 1.0, 0.7, and 0.5, respectively, for AZA1, −2 and −3. Those data differ from those proposed by EFSA and suggest the need for a reassessment for regulatory purposes (Table 4) [19]. Oral administration of AZAs showed that the main targets at the histological level were the liver, gastrointestinal tract, and spleen [215]. Toxicokinetic evaluation in mice after the acute oral administration of sub-lethal doses of AZA1 indicated that AZAs were readily absorbed and detected the highest amount of toxin in the liver, followed by kidneys, lungs, spleen, and heart, even though significant tissue damage was only observed at the intestinal level [222]. AZAs induce extensive damages to the gut including dilation and fluid accumulation in the small intestine, exfoliation of duodenal villi and infiltration of leukocytes [223,224]. However, from AZP symptoms, diarrhea has not been reported in mice, representing a hindrance to translate in vivo results to human poisoning [215]. Intraperitoneal injection of AZAs in mice induced the swelling of the liver, and histopathological analysis showed fat droplets in the hepatocytes cytoplasm and vacuoles in the centro-lobular and sub-capsular regions of the liver [225]. Liver, gastrointestinal, and lung damage was also reported by other studies after repeated oral exposure to sub-lethal AZA1 in mice even though the long-term effects are inconclusive [226]. Neurotoxic symptoms (spasms, a slow progressive paralysis) were also observed in mice treated with AZAs [227]. Intravenous or intraperitoneal injection of AZAs caused cardiotoxicity (arrhythmias, functional, and structural heart damage) and cardiovascular problems (altered arterial blood pressure) [214]. In addition to that, evaluation of toxins combination [87] along with their effects under chronic oral exposure [86] would support current public policies on consumers safety.

Marine animals are the main sources of AZA contamination, but only a few studies were performed in vivo with *Azadinium* revealing a negative effect on feeding behavior of mussels [228]. The toxic effects detected in mussels could be used as early indicators of contamination associated with the ingestion of seafood [229]. Additionally, a potential adverse outcome of AZAs in fish development was suggested, with consequent ecological impacts [230].

Only levels of AZA1, AZA2, and AZA3 are regulated in shellfish at international level as a food safety measure based on occurrence and toxicity [49]. The regulatory limit set by the European Union (EU) legislation for azaspiracids is 160 µg AZA eq/kg shellfish flesh, and the reference method for toxin monitoring in bivalve mollusks for human consumption is the analysis by LC-MS/MS [19,103].

Pectenotoxins (PTXs) take their name from the organism where they first were discovered: the digestive gland of Japanese scallop, *Patinopecten yessoensis*. These toxins are heat-stable polyether macrolide compounds, of which PTX2 is believed to be the main precursor originating other analogues during metabolic processes in bivalves [231]. They have been shown to cause cytoskeleton disruption by binding actin in vitro [2,70,180,232].

Up to last year, in the European Union, PTXs were considered in the same group of OA toxins for regulatory purposes with a limit of 160 µg of toxin equivalent/kg of shellfish meat (EU Regulation 853/2004) [103]. However, EFSA has concluded, in its Opinion on Marine Biotoxins in Shellfish—Pectenotoxin Group, that PTXs in shellfish are always accompanied by toxins from the okadaic acid group, and there are no reports of adverse effects in humans associated with PTXs [66]. Therefore, at present and based on EFSA opinion, PTXs have been removed from the list of marine biotoxins to be analyzed in live bivalve mollusks in Commission Delegated Regulation (EU) 2021/1374 [65]. Looking at the historical process that leads to the regulation of PTXs, PTXs were initially legislated since they might have been responsible for outbreaks of human illness involving nausea, vomiting, and diarrhea in Australia in 1997 and 2000 [233]; however, the symptoms were later attributed to OA esters [18]. In fact, the presence of these toxins in shellfish was discovered due to their acute toxicity in the mouse bioassay after i.p. injections of lipophilic extracts of shellfish [66]. Therefore, even though there are no reports of human illness causally associated with exposure to PTX-group toxins, PTXs remained regulated for decades until last year.

Yessotoxins (YTXs) are disulfated polyciclic ether compounds usually detected with OA toxins, so they were also included in this OA group initially [12]. Further on, they were reported not to share molecular targets, and their toxicological effects were not comparable. This resulted in yessotoxins being considered a separated group of toxins from OA [2]. YTXs are produced by the dinoflagellates *Protoceratium reticulatum*, *Lingulodinium polyedrum*, and *Gonyaulax spinifera* [2]. They enter in the food chain since they accumulate in edible tissues of filter feeding shellfish [234]. YTXs’ mechanism of action is not fully understood. It was suggested that it could involve cross-talks between cAMP, calcium, phosphodiesterases, protein kinase C, and A-kinase anchor proteins as well as mitochondria, where the role of each signaling alteration and the final effect depend on the cellular model [235]. Additionaly, modifications in second messenger levels, protein levels, immune cells, and cytoskeleton have been published as consequence of YTXs exposure [235]. In addition, YTX seems to be a cellular death inducer in some types of tumor cells [236,237,238], to exert a cytotoxic effect in neuronal cortical neurons [239], and to display apoptotic activity in the cortex and medulla of mice after intraperitoneal administration [240]. The lethality on mice after i.p. injection has been reported, but when YTX was given orally, no poisoning symptoms were developed, and there are no records about human intoxication events [12,70,235,241].

YTXs were included in the list of marine toxins regulated due to the coexistence with diarrheic toxins and the lethality on mice after i.p. injection [241]. The regulatory limit for YTXs was 1 mg yessotoxin equivalent/kg (EU Regulation 853/2004) [103]. However, in 2013 and in the light of the EFSA Opinion and of the conclusions of the 32nd Session of the CODEX Committee on Fish and Fishery Products, the European Union increased the limit for yessotoxins to 3.75 milligrams/kg of shellfish meat [242]. This seems to be a preventive measure, taken to avoid a possible poisoning if it is ingested at very high doses, since, as mentioned above, low oral toxicity has been reported, and there are no records of human intoxications [235].

**Figure 8:**
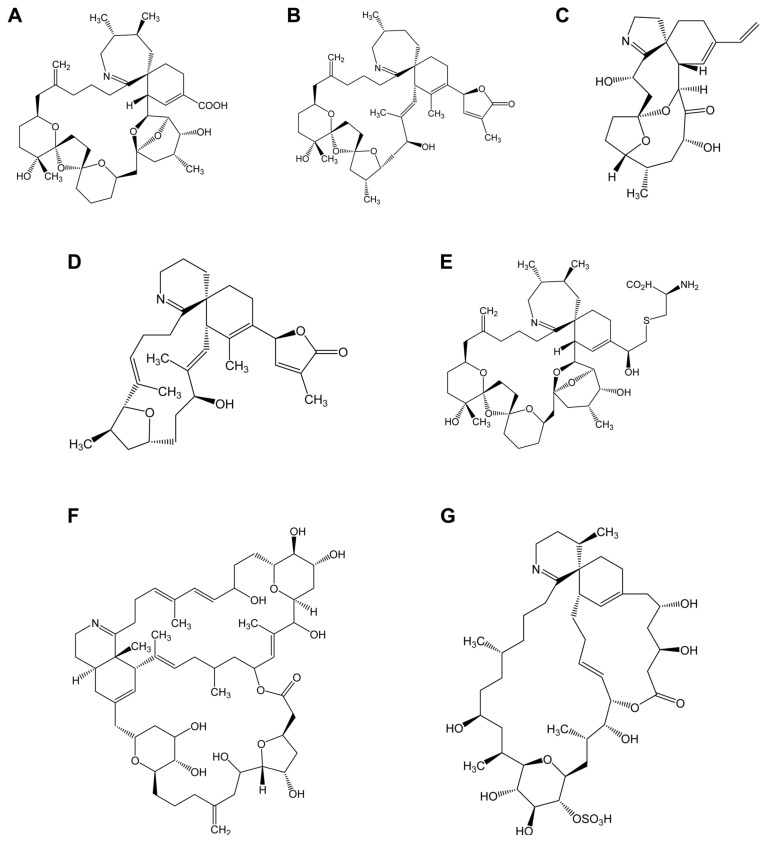
Chemical structure of the main cyclic imines: pinnatoxin A (**A**), spirolide A (**B**), portimine (**C**), gymnodimine A (**D**), pteriatoxin A (**E**), prorocentrolide (**F**), and spiro-prorocentrimine (**G**).

Cyclic imines (CIs) are a numerous group of more than 40 analogues, including pinnatoxins (PnTXs), spirolides (SPXs), gymnodimines (GYMs), pteriatoxins (PtTXs), porocentrolides (PcTXs), spiro-prorocentrimines, and portimine (Figure 8) [15,243]. They have been grouped together because of their common imine group as a part of a cyclic ring, which confers the pharmacological and toxicological activity, and due to their similar acute fast-acting toxicity in mice [244]. They are produced by several microalgal species such as *Vulcanodinium rugosum* and *Alexandrium ostenfeldii* and are distributed globally. CIs have been reported in algal samples from Scottish waters and in shellfish from Norway and the French Atlantic coast [243,245,246]. Their mechanism of action is understood and relies on the inhibition of nicotinic acetylcholine receptors (nAChRs) [247]. Five subunits assembly into a circular homopentamer or heteropentamer structure, constituting the ligand-gated ion channel [15,248]. Molecular simulations along with the crystallographic structure of CIs bound to acetylcholine binding protein and nicotinic receptor have greatly provided insight into their interaction [15]. CIs settle in the ligand-binding pocket placed in between two subunits. They directly interact with loop C [(+) face] aromatic residues of the first subunit and, to a lesser extent, with loop F [(-) face] of the following subunit [249]. They reversibly block these channels, impeding the transmission of neuronal- evoked ACh-mediated muscle contraction [248]. Variability in affinities for each nAChRs subtypes found in nerves and muscle may account for differences in potency. Indeed, the symptomatology described in vivo is in accordance with antagonism of these receptors. Following oral exposure, animals develop tremors, reduced mobility, hind leg paralysis, and jumping and breathing difficulty, which can result in death [15,248,250]. Supporting this statement, CIs have been shown to impair neuron-induced muscle contraction directly in the neuromuscular junction [251]. Regarding structural data, high-resolution binding of CIs to nAChRs would complete the understanding of their interaction and the conformational changes induced by these toxins.

Although cyclic imines (CIs) have been found to be highly toxic to mice, there is no evidence of intoxication in humans [15,243,252]. In 2010, the EFSA panel estimated exposure to spirolides did not present a health risk to shellfish consumers but that exposure risks from other cyclic imines could not be assessed [244]. Therefore, CIs are not yet regulated in Europe due to a lack of sufficient toxicological and epidemiological data needed to establish health safety thresholds. Even though no information has been reported yet linking CI toxins to neurotoxic events in humans, the potent interaction of PnTX with central and peripheral nAChRs raises concerns about the harmful downstream effects of PnTX exposure. In recent years, there has been increasing evidence for the occurrence of emerging PnTXs in various wild or commercial shellfish species, collected at different periods of the year, and in different marine waters. Related to that, the levels of PnTXs in shellfish at the Mediterranean Ingril lagoon in France were much higher than those reported in contaminated shellfish from other locations such as Norway, Spain, or Canada [253,254]. Given the potent antagonism of PnTXs against muscle and neuronal nicotinic acetylcholine receptors, a risk for human consumers may exist when PnTX shellfish accumulation reaches high levels [248,255,256]. Therefore, in 2019, the French Agency for Food, Environmental, and Occupational Health and Safety established a limit value of 23 μg PnTx-G/kg of total shellfish meat [255,257].

In conclusion, YTXs are legally regulated in Europe, even though there is a lack of demonstrated toxic effect in humans; PTXs remained regulated up to 2021, although they are nontoxic to humans, while CIs, which were reported to be neurotoxic and frequently detected in shellfish, are not regulated. This highlights the urgent need to pay attention to the update evidence versus the old spread knowledge in marine phycotoxins (Table 5) and to harmonize regulatory criteria about all these toxins.

Future challenges related with lipophilic phycotoxins (included in Figure 9):

- Well-characterized epidemiology studies;
- Further understanding of the molecular target of phycotoxins;
- Review the mechanisms of phycotoxins toxicity;
- Establishment of objective toxicity parameters to determine accurate TEF values;
- Advances on the knowledge of oral toxicity;
- Health effects associated to the chronic exposure of phycotoxins and studies of repeated exposure to toxins at levels below the current regulatory limit;
- Elucidation of the mechanisms of phycotoxins biotransformation in seafood and in the human body;
- Improvements in the information regarding toxicokinetics (i.e., absorption, distribution, metabolism, and elimination);
- Increase of data concerning the bioactivity of different toxins relevant to the assessment of toxicity;
- Further research on the effects of toxins mixtures;
- Identification of the impacts of phycotoxins on marine animals;
- Common legislative criteria: toxin regulation, implementation of effective toxin monitoring, and management programs for toxins.

### 3.4. Toxins Acting on Ion Pumps: Palytoxins, Ostreocins, and Ovatoxins

PLTXs are a group of more than 25 compounds sharing a complex polyketide structure [2,86,258]. Among analogues, ostreocins, ovatoxins, and mascarenotoxins are found along with homoPLTX, 42-hydroxyPLTX, bishomoPLTX, neoPLTX, and deoxyPLTX [86,258]. PLTX binds to the Na^+^/K^+^ ATPase pump, stabilizing an opening conformation that allows the flux of cations following their concentration gradient; thus, the pump is converted into a non-selective ion channel (Figure 2) [2,259]. Na^+^/K^+^ ATPase actively transfers Na^+^ and K^+^ ions against the concentration gradient, maintaining ion homeostasis between extracellular and intracellular media. Modulation of its physiological activity can lead to severe cell and tissue impairment. The structural binding of the toxin to Na^+^/K^+^ ATPase pump is currently unknown. It is hypothesized that PLTX binds to the extracellular region of the protein, since its effects are only observed when applied extracellularly [259]. The ion imbalance caused can trigger depolarization of neurons and muscle cells [2], which is in accordance with the symptomatology resulting from PLTX poisoning. Currently, data about PLTX binding structure to Na^+^/K^+^ ATPase pump, as well as its analogues, are of great interest in the understanding of the PLTX mode of action.

Palytoxin intoxication may occur after physical contact with contaminated water (bathing activities) or inhalation of the marine aerosol containing PLTX as well as the consumption of contaminated seafood [260]. PLTX compounds threaten human health and marine life and can have an impact on tourism (beach closures), commercial fisheries, and aquaculture [261]. PLTX is heat-stable and is not eliminated by normal cooking or boiling. Therefore, PLTX poisoning is mostly related to the ingestion of PLTX-contaminated seafood and involves mainly respiratory, skeletomuscular, cardiovascular, gastrointestinal, and nervous symptoms [262]. Symptoms associated with PLTX ingestion depend on the toxin concentration and comprise a bitter and metallic taste, paresthesia, myalgia, hypertension, nausea, abdominal cramps, vomiting, diarrhea, cardiac dysrhythmias, hemolysis, respiratory distress, renal failure, and coma, which can lead to death in the most severe cases [2,86,263]. However, reliable quantitative data on acute toxicity in humans are unavailable [111]. Several cases of respiratory poisoning, skin injuries, or ocular exposure have been reported in beachgoers due to aerosols released during massive blooms of *Ostreopsis* [260]. Poisoning was also reported in aquarium hobbyists from incidental contact with PLTX-producing *Palythoa* [264]. The most common signs after inhalation and cutaneous exposures are respiratory distress, bronchoconstriction, mild dyspnea, rhinorrhea, cough, fever, and a small incidence of dermatitis and conjunctivitis [265].

The toxicity studies performed on a few PLTX congeners showed that, despite the small diversity in structure or even in stereo-structure, their relative toxic potencies might be quite different either in vivo or in vitro [262]. Palytoxin is highly neurotoxic and increases the cytosolic calcium concentration while decreasing intracellular pH in neurons [266]. The membrane depolarization generated and the massive increase of Ca^2+^ in the cytosol interferes with some vital functions [263]. Palytoxin triggers a series of toxic responses; it inhibits cell proliferation and induces cell rounding, detachment from the substratum, and F-actin disruption [267]. Recent in vivo evaluation of PLTX indicates a high chronic toxicity with a lower NOAEL than previously determined for acute toxicity, which pointed out the need to consider this toxicity in risk assessments.

PLTX has been shown to be harmful and occur in EU while it is not regulated [36,37]. An ARfD of 0.2 μg/kg (sum of palytoxin and ostreocin-D) has been established through experimental toxicity data [268]. PLTX is extremely potent through intravenous, intraperitoneal, and intratracheal exposure. However, in mice, the oral toxicity of palytoxin is found to be three times lower than the i.p. toxicity with LD_50_ 651–767 ng/kg. This is because palytoxin absorption is less efficient through the gastrointestinal tract than through the peritoneum due to the molecular weight and hydrophilia [269]. Biochemical changes were observed following oral administration of palytoxin in mice, and histological changes include inflammation in the forestomach [270,271]. A cardiac damage was supported by the in vitro effect of the toxin on cardiomyocytes [272]. Palytoxin topically applied to the skin or eyes cause skin irritation and erythema in mice [273].

Treatment is symptomatic and supportive. In case of ingestion of PLTX-bearing seafood, treatments, such as gastric lavage, fluid administration, forced diuresis therapy, and artificial respiration, are applied [274]. Victims recover after a few hours to days. To prevent dermal or inhalational exposure in persons handling zoanthids, they should wear gloves and a breathing mask.

Today, PLTX is considered one of the most toxic non-protein natural compounds. Additionally, there are increasing records of PLTX presence in many edible marine organisms from the European coasts [275]. The efficiency of risk assessment of PLTX relies on the evaluation of this phycotoxin in fish and shellfish as well as adequate research, more relevant in the case of a toxin that demonstrates a potential higher health risk. However, the EU still has not adopted a maximum permissible limit to confront the risk of PLTX poisoning.

Future challenges related to lipophilic phycotoxins (compiled in Figure 9):

- Studies to assess the real hazard they present to humans;
- Detailed epidemiological studies are needed to better evaluate safety levels and to promote regulations that will protect human health and reduce economic losses;
- Pharmacological and toxicological effects of each PLTX analogue to carry out reliable structure–activity relationship;
- Evaluation of PLTX and analogues oral toxicity;
- Studies exploring treatments for PLTX including search for effective antidotes;
- Common legislative criteria: toxin regulation, implementation of effective toxin monitoring, and management programs for toxins.

## 4. Marine Toxins as a Source of Drugs

Phycotoxins not only cause damage but also have therapeutic applications based on their specific interactions with their natural targets, converting these compounds in natural sources to develop new drugs. The diversity in their mechanisms of action pointed to these substances as lead compounds for the discovery of new drugs. However, the research in this field has not yet been fully developed [276].

Despite the great potential of these compounds as therapeutic agents, the development of new drugs from marine toxins is complex due, among other reasons, to the low availability of these biological products and the difficulty of obtaining them in sufficient quantities [277]. In the next paragraphs, some of the marine phycotoxins with potential clinical applications are summarized.

Tetrodotoxin (TTX) is a neurotoxin with one of greatest potentials to be used for therapeutic purposes due to its potent effect blocking the voltage-gated sodium channels preventing nerve and muscle function and consequently, the transmission of the pain signalling [84]. Since Na_v_ isoforms distribution in the nervous system is different [278], TTX is not effective in controlling all the different types of pain. Many studies have been performed using TTX as an agent to relieve different types of pain, administering TTX at different doses and by different routes [279,280,281,282]. Among these uses, the best results have been obtained in preclinical and clinical studies for alleviation of neuropathic pain [279,283]. Inflammatory pain and acute pain have also been evaluated in preclinical studies. Although the data obtained are still unclear, they suggest that the administration of TTX might have little impact on these types of pain [282,284,285]. More studies are needed to evaluate the benefit of the administration of this toxin to control different types of pain.

TTX efficacy in relieving pain associated with cancer has also been evaluated with inconclusive results. However, data published to date indicate that TTX is a good tool of research for therapeutic purposes against opioid-resistant pain in cancer patients, with reported mild to moderate adverse effects, which are generally transient [286] and well tolerated at therapeutic doses, even when TTX was administered for a long period [287,288]. In fact, a promising drug containing TTX as the main active compound for the management of cancer-related pain is Halneuron^®^ (Vancouver, Canada Wex Pharmaceuticals Inc.), currently into Phase III clinical development for the treatment of cancer related pain and also in Phase II clinical trials development for chemotherapy-induced neuropathic pain https://wexpharma.com/technology/about-halneuron/, accessed on 11 January 2022, with a considerable number of patients obtaining good and promising results [283].

The blocking effect of VGSC caused by TTX has also been investigated for its potential as local anesthetic, with the advantage that TTX is extremely potent and cause minimal local toxicity [289]. It has been demonstrated that TTX provided a prolongation of the duration of local anesthesia without significant systemic or local toxicity when combined with an eluent, thus maintaining a sustained release at a sub-therapeutic level and enhancing the effects of the anesthetics [289]. Additionally, significant improvement in the efficacy of the analgesic effect induced by TTX was reported when co-administered with vasoconstrictors or well-known local anesthetics [290,291]. The best results were obtained when administrating the anesthetic encapsulated in microparticles in combination with TTX [292]. Therefore, there are commercial formulations containing TTX under development for local and topical anesthesia [293]. Despite all these potential promising therapeutic applications of TTX, it must be taken into account that TTX has a very narrow therapeutic window, which means that TTX release must be controlled to avoid systemic toxicity [289].

Similarly to TTX, STX and its analogues have a therapeutic potential as anesthetic agents since they are highly selective sodium channel blockers [99]. Actually, there are several research reports that support the therapeutical use of STX and its analogues regarding their potential in pain management [294], even in combination with other known pain modulators, increasing their efficacy and potency without exacerbating their toxicity [290,294,295,296,297].

OA and its analogues could be research tools for future investigations and useful probes for the discovery of new neurodegeneration and cancer drugs since the activity of serine/threonine protein phosphatases is a potential target for novel therapeutics with applications in many diseases including cancer, inflammatory diseases, and neurodegeneration [298,299]. Furthermore, OA has been shown to possess fungicidal and antimicrobial activity since it has been reported that it inhibits the growth of *Candida albicans*, *Aspergillus niger*, and *Penicillium funiculosum* [300].

The potential therapeutic use of yessotoxin and its derivatives has been postulated for Alzheimer’s disease using in vitro models [301] and for treating and/or preventing metabolic diseases [302], a controversial fact due to the neurotoxicity of this substance, as well as being an antiallergic compound [238,301]. Moreover, the effect of yessotoxin inducing apoptosis of tumoral cells in certain types of tumors has been reported in several studies, suggesting that this toxin could be a compound with great potential to develop future antitumor therapies [235,236,238,302,303,304].

Although, so far, maitotoxin was historically considered one of the most toxic natural compounds, its range of in vivo toxicity described in the literature varies from 50 to 200,000 µg/kg [140,305,306,307,308,309]. This is an important fact that needs to be clarified since there are several effects of MTX on cellular regulatory mechanisms, which could make it a molecule with potential use for different purposes, for instance, neurotransmitter secretion [310], programmed cell death activation [311], fertilization [312], and insulinotropic activity since it has been reported that MTX activates non-selective cationic currents (NSCCs) [313,314], becoming a promising new tool available for the development of multiple new therapies for different purposes.

13-desmethylspirolide C has been reported to have beneficial in vitro and in vivo effects against neurodegenerative diseases, decreasing the amyloid beta load and Tau hyperphosphorylation in in vitro experiments with primary cortical neurons and an in vivo murine Alzheimer’s disease model [315,316]. Another recent in vitro study has confirmed the neuroprotective effect of 13-desmethylspirolide C on human neuronal differentiation using a human neuronal stem cell line [317]. All these findings make SPXs and related compounds attractive molecules for the development of new therapies against neurodegenerative disorders [316,317].

Azaspiracids’ blocking of hERG potassium channels suggests that they could be used as antiarrythmic drugs [212] since the hERG channel has been shown to be the target for class III antiarrhythmic drugs, which can reduce the risk of re-entrant arrhythmias by prolonging the action potential duration and refractory period without slowing the conduction velocity in the myocardium [213]. Moreover, since there are not selective extracellular activators of VRACs besides the intracellular application of the GTPγ-S (guanosine 5′-O-[gamma-thio]-triphosphate) molecule, this group of natural compounds could represent a way to analyze the role of these channels in cellular homeostasis. This fact is important since VRAC inhibitors have proven to be useful to modulate cancer progression, preventing the transition of tumoral cells to phase S of the cellular cycle [318]. Therefore, these marine toxins can be useful in vitro to test the anticarcinogenic effect of compounds, enabling concise and precise description of the experimental results, their interpretation, and the experimental conclusions that can be drawn.

Gambierol and its analogues are compounds with great potential for drug development owing to their main target, voltage-gated potassium channels (K_v_). The different K_v_ subunits play a critical role in cellular homeostasis regulations; thus, each K_v_ subunit is involved in a certain pathology. In this sense, K_v_1.1 inhibition has been reported to be implicated in pain sensation, becoming an important target to develop anesthetics [319]. In addition, K_v_1.2 blockers are important in the treatment of multiple sclerosis [320]. Moreover, K_v_1.3 and K_Ca_3.1 channels inhibitors have potential uses in immune responses [119,321], multiple sclerosis [322], rheumatoid arthritis, and type I diabetes mellitus [323], thus becoming a good tool to develop immunosuppressive therapies [324] and for neurodegenerative diseases [325]. K_v_1.4 is important in the management of diabetes preventing some biochemical abnormalities [326], and K_v_1.5 is important for cardiac excitability [327]. Therefore, gambierol could be a very promising compound from which to develop drugs for the treatment of multiple pathologies; however, the potential of this marine compound has not been yet developed and more studies and research are needed.

## 5. Detection Methods

Detection methods for marine toxins that threaten human health are needed to warrant food safety. Different approaches to detecting these compounds have been addressed by the scientific community, all of them having advantages and disadvantages that make them suitable for specific purposes. In general, marine toxin-detection techniques can be classified into two groups: analytical and non-analytical methods. Non-analytical methods could be subsequently subclassified into molecular interaction/activity-based assays, cell-based assays, and animal bioassays. Considering the characteristics of the technique, some will be adequate as confirmatory methods to identify and accurately quantify toxic molecules, while others may be useful for rapid sample screening or even on-site toxin detection and others may be useful as sentinels for yet unknown or new toxic activity.

### 5.1. Analytical Detection Methods

Analytical methods allow the identification and quantification of toxic molecules, as far as an analytical standard is available for reference. Most analytical methods for marine toxins are based on liquid chromatography (LC) separation followed by a detection technique. With regards to LC, reverse-phase high-pressure liquid chromatography (HPLC) and ultrahigh-pressure liquid chromatography (UPLC) have been used extensively. LC has been coupled to fluorescence, light absorbance, or mass spectrometry detection, depending on the targeted toxic compounds. Among mass spectrometry techniques, tandem mass spectrometry (MS/MS) with multiple reaction monitoring (MRM) mode is commonly used for routine detection and has gained remarkable relevance, especially for lipophilic toxins, in the last decades. MS/MS working in MRM mode is a targeted method, meaning that it searches only for specific toxins in the sample. There have been some attempts to develop non-targeted screening of toxins using high-resolution mass spectrometry; however, at the moment, the proposed methods provide only an extended targeted method or an unpractical alternative that requires fully matched matrix controls [328].

Because of the characteristics of analytical methods, their validation is relatively easy and, therefore, several of them have become official or reference methods for marine toxin detection. Although there are multiple analytical alternatives, the most commonly used for routine detection are those that have been officially validated.

The first analytical method validated for marine toxins was domoic acid detection by HPLC coupled to ultraviolet light absorbance (HPLC-UV) by the AOAC [329]. Not long after, HPLC coupled to fluorescence detection (HPLC-FLD) was published as an AOAC official method for the detection of PSP toxins in 2005 [330]. However, this PSP-detection method required precolumn derivatization of some toxins for adequate identification of the PSP toxin profile of a sample, which is laborious and, therefore, requires significant hands-on time of laboratory personnel. Later developments significantly improved the HPLC-FLD detection of these toxins using a similar approach with postcolumn derivatization, which was also validated [331]. Currently, TTX detection simultaneously with PSTs has been achieved with LC-FLD [332].

LC-MS/MS provides efficient detection of lipophilic toxins simultaneously in the same sample, including OA and DTXs, AZAs, pectenotoxins, and yessotoxin, and was fully validated for mollusks by the European Union Reference Laboratory [333] and the European Committee of Standardization [334]. Because, in the case of lipophilic toxins, LC-MS/MS detection offered a great improvement in performance for seafood monitoring purposes compared to previously used methods, it displaced them in routine testing laboratories. The flexibility of MS/MS in detecting multiple compounds, once the technique was available in many laboratories, prompted the use of LC-MS/MS to detect other toxin groups such as domoic acid and PSP toxins. PSP detection by LC coupled to tandem mass spectrometry (LC-MS/MS) has been optimized and offers the advantage versus LC-FLD of no sample derivatization needed to identify the different analogues of this toxin class [335,336,337], including simultaneous detection of TTX in some protocols [338]. UPLC-MS/MS detection has also been validated for domoic acid [339]. Integrated analysis of regulated lipophilic and hydrophilic toxins in a single LC-MS/MS protocol can be achieved by the optimization of LC conditions [340]. However, simultaneous detection of these toxins in one single sample requires a unique sample preparation protocol for PSP toxins, DA, and lipophilic toxins (OA, DTXs, AZAs, PTX, and YTX) that is not available yet.

Minimum performance requirements of these methods to allow adequate detection and quantification below current maximum regulated limits have been internationally established to warrant human safety [49].

Alternatives to LC for toxins separation previous to coupling to mass spectrometry have also been tested, such as capillary electrophoresis (CE-MS/MS) for hydrophilic toxins [341], but they are far from being routine monitoring techniques.

Adequate evaluation of sample toxicity with analytical methods requires considering analogue toxic potency within the toxin class, which is achieved by application of TEFs. TEF values for several analogues of the PSP, DSP, and AZA groups have been estimated and published by FAO [70]. However, reliable estimation of TEF values is still missing for many analogues of most marine toxin groups.

In LC-MS/MS procedures for lipophilic toxin detection, besides OA, DTXs and AZAs, cyclic imines, and yessotoxins are often analyzed [333,342,343]. Detection of palytoxins and ciguatoxins by LC coupled to high-resolution mass spectrometry has also been achieved in research laboratories and some monitoring laboratories [344,345,346], but results evaluation is more difficult due to the lack of commercial certified reference standards of these toxins and the scarcity of toxicological information to determine TEFs, and therefore they have not become widespread routine testing methods for these toxin classes yet. In addition, high-resolution mass spectrometry has lower sensitivity than MS/MS, which is not adequate for identification of these toxins [347].

Sample preparation is usually a critical step in marine toxin monitoring, mainly in food samples, in which complex matrixes are an important source of interference. Recent advances in sample cleaning protocols incorporated the QuEChERS procedure, a combination of extraction with solvents and dispersive solid-phase (dSPE) extraction that improves preanalysis clean-up and, as a result, also LC-MS/MS general performance and sensibility [348,349].

Analytical methods require expensive instrumentation and qualified personnel and, therefore, they are not suitable for on-site detection by food handling end users. On the other hand, although they are not considered particularly fast, recent works have demonstrated that they are amenable for automated sample analysis [350,351]. One of the main disadvantages of analytical methods is that the lack of reliable TEF values for every analogue of each marine toxin group makes them unsuitable for quantification of sample toxicity. In addition, adequate identification and quantification of every toxic molecule require certified reference standards, which are not commercially available for many analogues of marine toxin groups, with remarkable problems related to this issue when considering ciguatoxins and palytoxins. Finally, they are targeted techniques that only search for the programmed molecules but would miss any other toxin. In spite of the widespread use of analytical techniques for marine toxin detection in routinary seafood monitoring, owed to the characteristics discussed above, these methods do not provide optimal protection of seafood consumers.

### 5.2. Molecular Interaction/Function-Based Assays

Many methods developed for marine toxin detection are based on the specific interaction of the toxin with a molecule, often a macromolecule. These assays do not usually allow the identification of the toxic molecule, because they target a toxin class and cannot detect different analogues of the same group independently. Owing to this important characteristic they are not suitable for accurate quantification of individual toxins, and they should be considered as semiquantitative methods useful for estimation of sample toxicity. Only a few of them have been officially validated for toxin detection.

In most cases, the assay measures the interaction of the toxin with a specific binding protein, but modification of the protein function has also been used as a reporter of toxin presence. Although receptors could be used, the most common binding proteins are antibodies. In spite of the difficulty of producing antibodies specific for small toxins, many different immunoassays have been developed for the detection of these molecules. Immunoassays are easy-to-use, robust techniques that can be performed in the laboratory or adapted to on-site screening. ELISAs for several marine toxins, including OA and DTXs, AZAs, DA and PSTs, and even palytoxin and ciguatoxins, have been developed for detection in the laboratory, usually through indirect or competitive designs [352,353,354,355,356,357]. Actually, one of the official methods for DA detection is an ELISA acknowledged as an official method for screening purposes in many countries [330]. The excellent performance of antibodies as binding partners granted their use in different technological developments that can make toxin screening more efficient and even adapted to on-site detection, such as several immunosensor devices [358,359,360]. Lateral flow immunoassays (LFIA) are suitable for unexperienced end-users and have been used for multiple applications. LFIAs have also been adapted to marine toxin detection, including PSP toxins, OA and DTXs, being commercially available for a while even in a dipstick form [361,362,363,364]. In addition, immunoassays are easily adapted to high-throughput screening [365,366]. Multiplexing by combining several immunoassays for different analytes performed simultaneously in the same sample can be achieved by microsphere-based flow cytometry-like detection, high-density spotting microarrays, or parallel or in-line processing in biosensors, as it was demonstrated for PSP toxins, the OA group, and DA [359,367,368].

One of the main disadvantages of immunoassays is that antibodies are not specific for a toxin but rather can interact with several analogues of the toxin class. Consequently, toxic molecules cannot be independently identified. In addition, crossreactivity among analogues rarely matches toxic potency. Therefore, the final results will be affected by the toxin profile of the sample, and they should be considered an estimate of sample toxicity, not an accurate quantification. There are, though, some exceptions, such as a DSP toxins antibody whose crossreactivity for OA and DTXs fairly matches toxic potency [369].

There are a few techniques that employ other binding molecules. Receptors, usually the macromolecular natural targets of the toxins, have also been used for marine toxin detection assays, although they are not as robust as immunoassays owing to protein stability issues. Because receptor-based assays utilize toxin targets for detection, they bind to several analogues of the toxin class, not allowing identification of toxin molecules. However, the affinity of the toxin analogues for their natural target would be a better indicator of toxic potency than antibody affinity. Several receptor-binding assays have been published for marine toxins, one of them being an official method for the detection of PSP toxins [370,371,372,373]. An important drawback of this official assay is the use of radioactivity. Similarly, a receptor-based assay has been developed for ciguatoxins based on competition with radioactively labeled brevetoxin; however, in this case, an alternative non-radioactive approach using fluorescently labeled brevetoxin has been subsequently produced and is commercially available [347,372], although not specific for ciguatoxins, because the result would be affected by the presence of other sodium channel binding toxins such as brevetoxins. A nicotinic receptor-based assay was also developed for the detection of cyclic imines and adapted to different readout techniques [373,374,375,376,377].

An interesting variation of receptor-based assays are those that measure modification of target activity by the toxin. The protein phosphatase assay for OA and DTXs is an excellent example of this assay type. The initial detection strategy used conversion by PP2A of a substrate to a colored or fluorescent product in a microplate detection design [378,379]. Innovative approaches have adapted this enzyme inhibition assay to electrochemical sensors as biosensing devices [380,381]; however, their adequate performance in food samples has not been demonstrated.

Overall, immunoassays and receptor/target-based assays are fast, sensitive, and cost-effective, and some of them have been adapted to portable or on-site assays and to high-throughput techniques. These assays should be considered semiquantitative methods useful for rapid screening of food samples, and further development and validation should provide testing assays needed to warrant reliable on-site detection of all regulated toxins. Probably, considering progression in the last decades, LFIA is the most promising detection technology to achieve efficient on-site detection for marine toxins in the near future, although antibody crossreactivity profiles for most groups are still an obstacle for adequate screening and must be improved. Currently, for some specific toxins, such as ciguatoxins, receptor-based assays offer great possibilities. One of their main advantages is a better relationship between toxin detection ability and toxicity, and future technical developments will probably allow the integration of receptor-based methods in on-site detection procedures.

Besides receptors, enzymes, and antibodies, aptamers, a relatively new class of recognition biomolecules, have also been explored for marine toxin detection. Aptamers are synthetic single-stranded oligonucleotides, either DNA or RNA, with stable three-dimensional conformation that confers specific binding to analytes with high affinity and selectivity [382]. One of the advantages of aptamers is that they can bind molecules for which it is difficult to produce antibodies, such as highly toxic or small compounds that do not trigger an immune response, as it is the case of most marine toxins. Specific aptamers for STX, GTX1/4, DA, OA, tetrodotoxin, and palytoxin, among others, have been published and integrated for toxin detection in sensors using different transducer technologies (Table 6). Some of them have shown remarkable sensitivity and compatibility with shellfish extracts (Table 6); however, aptamers used in these assays do not bind other analogues of the toxin class [383,384,385,386,387]. Detection of only one analogue of a toxin group does not provide adequate protection, and extended use of aptamers for marine toxin monitoring in seafood will have to cover, at least, the more frequent toxic analogues. Interestingly, this approach will allow identification of different analogues of the group. Considering that simultaneous detection of two toxins has been already demonstrated [384], it may be extended in the future to more molecules. Although much work is still needed in this field for efficient on-site evaluation of overall sample toxicity, recent developments make aptasensors a promising technology for marine toxin detection, because they are easy-to-use, portable, sensitive methods that use synthetic, low-cost, stable sensing molecules. Although a great effort has been made in the last years in this field, it is still to be demonstrated if aptasensors can provide efficient on-site detection for marine toxins. For this purpose, evaluation of overall sample toxicity with aptasensors based on specific aptamers for each toxin would depend on the availability of adequate TEFs as much as analytical methods do. Therefore, a fair estimation of toxicity would not be possible at the moment, even if other technical issues were solved.

### 5.3. Cell-Based Assays

Cell-based assays are widely used to study human and animal diseases and design therapeutic strategies [397]. However, they can also be used to determine if marine toxins are present in a seafood or water sample. There are many types of cell-based assays since a toxic agent may cause cytotoxicity via different mechanisms. Hence, when designing these methods, it is important to choose adequate cell types (primary cell, native or engineered cell-line, species of origin…), reporters, number of repetitions, and data analysis strategies for reliable results [398]. Cell-based assays usually offer high sensitivities and are amenable for automation [398]. In addition, in vitro cell assays can be performed with human cell types, providing information relevant for human species, as opposed to in vivo bioassays in animals. Nevertheless, other factors such as cost, speed, and trained personnel and instrumentation requirements do not favour routine use for toxin detection.

Today, the most-used cell-based assay to detect marine toxins is the viability assay, which consists of determining the number of healthy cells exposed to a sample for an incubation period. To make it possible, several viability tests can be employed, such as dye exclusion, colorimetric, fluorescent, or luminescent assays. One of the most popular tests, as thousands of published articles evidence, is the MTT (3-(4,5-dimethylthiazol-2-yl)-2,5-diphenyltetrazolium bromide) assay. This assay allows determining the mitochondrial function through the metabolization at 37 °C of this tetrazolium salt to formazan dye by dehydrogenase enzyme, present in viable cells [399].

Viability assays have been used to detect OA, DTXs, AZAs, PSTs, palytoxin, and ciguatoxins (Table 7) [400,401,402,403,404]. Yet, some toxins need an addition of other compounds to show the cytotoxic effect, as it is the case of ouabain and veratridine to show the cytotoxic effect of saxitoxin [403]. A variation of this kind of assays is the hemolysis assay for palytoxin detection in microplates [405].

Remarkably, a cell-based cytotoxicity assay is being used for screening the presence of ciguatoxin in fish samples using the neuroblastoma cell line Neuro-2a, a co-treatment with veratridine and ouabain and MTT readout [347,406,407].

Other cell-based assays, such as electrophysiological assays [408,409] or measurement of membrane potential changes by fluorimetry [410,411], have been described for the detection of paralytic toxins with high sensibility. Recently, a new approach was proposed to detect saxitoxin or lipophilic toxins by identifying the changes in gene expression they produce in neuroblastoma cell lines or Caco-2 using qRT-PCR [412,413]. Nonetheless, even though these methods offer adequate detection, they are complicated to perform and not practical for routine food testing.

Cell-based assays do not allow the identification of analogues of a toxin group, sometimes not even of different groups, lacking the specificity of other methods. Therefore, they would provide an overall estimation of sample toxicity. In addition, portability for on-site analysis has not been fully resolved.

**Table 7:** Cell-based assays for some marine toxins.

Marine Toxin	Exposure Time	Cell Line	Detection Method	LOD *(IC_50_)*	Reference
OA DTX-1, DTX-2	24 h	V79 cells	MTT assay	*(27 nM)*	[414]
	48 h	HepG2 cells	MTT assay	*(30.2 nM)*	[415]
	24 h	Neuro2a cells	MTT assay	*(11.20 nM)*	[203]
AZAs	48 h	HepG2 cells	MTT assay	*(4.3 nM)*	[415]
PSTs	24 h	Neuro2a cells	MTT assay	0.91 nM *(8.6 nM)*	[403]
	24 h	NG108-15 cells	MTT assay	4.2 nM *(8.2 nM)*	[403]
	24 h	Neuro2a cells	Electrical impedance	0.1 nM	[409]
	24 h	Neuro2a cells	qRT-PCR	17 nM	[412]
	15 min	BE(2)-M17 cells	Fluorimetry	4.36 nM	[410]
	Immediately	Rat cortical neurones	Spectrofluorimeter	1 nM	[416]
CTXs	20 h	Neuro2a cells	MTS assay	*(0.02 nM C-CTX-1)*	[417]
PLTXs	24 h	Erythrocytes	Spectrometry	0.37 pM	[405]
	24 h	BE(2)-M17 cells	Spectrofluorimetry	0.07 nM	[401]

*MTT: 3-(4,5-dimethylthiazol-2-yl)-2–5-diphenyltetrazolium bromide; MTS: 5-(3-carboxymethoxyphenyl)-2-(4,5-dimethyl-thiazoly)-3-(4-sulfophenyl) tetrazolium; qRT-PCR: Real-Time Quantitative Reverse Transcription.*

### 5.4. Animal Bioassays

Mouse and rat bioassays have been the official methods for the detection of marine toxins in many countries for many years. These bioassays consist in the administration of a seafood extract to an animal, either by intraperitoneal injection or orally, and the observation of sickness symptoms or time to death. The mouse bioassay for the detection of PSP toxins has been validated by the AOAC and is still an official method for PSP monitoring [50]. However, due to ethical and technical issues, animal bioassays have been replaced in the last decades by analytical or other non-analytical methods with regards to routine toxin detection of lipophilic toxins [62] or as reference method for PSP toxins [418,419]. Despite the reduced use of these techniques, they are still useful tools as sentinels for new unknown toxins that may threaten human consumers.

Future challenges related to marine toxin detection (Figure 9)

- On-site, easy-to-use, efficient methods for detection of multiple toxin groups are not yet available;
- Certified analytical standards of some toxin classes are urgently needed;
- Improvement of sample preparation procedures for further testing or extended automation of routine monitoring;
- Reliable TEF estimation for many analogues of these toxin groups is still missing;
- Improvement of performance of analytical methods, especially for ciguatoxins and palytoxins.

## 6. Climate Change Uncertainties

Climate change is expected to have significant influences on both water quantity and water quality by shifting precipitation patterns, melting snow and glaciers, raising temperature, and increasing the frequency of extreme events. Phytoplankton responses to ongoing and future environmental change will significantly affect earth system processes at many scales. These primary producers are the photosynthetic base of marine food webs and responsible for approximately half of the global oxygen production [420]. Rising water temperature is likely to promote the spread and growth of microalgae in the sea [421]. Besides the beneficial impact of microbial eukaryotes, some of them can form HABs [9]. Global warming causes a decline of sea ice cover and an increase in the sea level, changing the physicochemical characteristics of the affected regions and impacting the ecology, the environment, and aquatic organisms at all trophic levels and potentially increasing the risk for future HABs even further [421,422,423]. The spread and increase of HABs worldwide is widely reported. Despite the extensive efforts to characterize the effects of climate-related environmental variables on different harmful dinoflagellates, it remains a challenge to predict their response to climate change and assess potential consequences related to their toxicity [424]. Climate change could be related to new toxins appearing in areas or products where they previously had not occurred, and new guidelines are needed about how to manage them. In agreement with that, the presence of tropical species such as Gambierdiscus, Fukuyoa, and Ostreopsis in temperate regions has been recorded. This fact constitutes a serious threat to human health by CTXs and PLTXs intoxications in the future.

Species of the genus Ostreopsis associated with PLTXs were first reported in Hawaii and Japan but are currently distributed worldwide, and blooms have been detected in the Mediterranean coast of countries such as France, Greece, Italy, and Spain [425]. Different PLTX-like compounds have been identified in the Mediterranean strains of O. cf. ovata and O. fattorussoi [60,426,427]. Currently aerosols with ostreocin are a problem in Mediterranean beaches. Blooms of Ostreopsis were also found on the coast of Portugal and the north of Spain indicating that species capable of producing PLTX analogues may be spreading from the Mediterranean to the north Atlantic [428]. Temperature seems an important factor determining both growth potential and toxin production of the genus Ostreopsis. Temperatures of 26–30 °C stimulated O. ovata cell growth and biomass accumulation and low toxicities, while temperatures of 20–22 °C induce higher toxicity per cell and cell numbers [111].

Ciguatera is endemic in certain tropical regions of the world but now is an emergent risk in Madeira and fish of the Canary Islands and Madeira, with a persistent incidence and impact on public health [429]. This northern expansion has been attributed to changes in distribution of toxin-producing microalgae. In support of that, the primary causative species of the genus Gambierdiscus and Fukuyoa have been recorded in the Canary Islands but also in the Mediterranean Sea. In fact, Gambierdiscus was detected in the Balearic Islands, being the northernmost point of this microalgae distribution worldwide. All these findings suggest a possible future concern about Ciguatera in finfish originating from Europe [430,431].

TTXs have been usually associated with contaminations of pufferfish in Japan; however, a recent emergence of TTX in different pufferfish, marine gastropods, and bivalve mollusks collected from Mediterranean countries has been reported [54,108,432,433]. Significant TTX levels have also been found in bivalves from northern latitudes such as England or the Netherlands and in shellfish harvested in the Atlantic coast of Spain and Portugal [107,434,435,436]. These can be linked to the increase of the presence of TTX-vectors, such as pufferfish species of the Tetraodontidae family, in these waters due to the increase in global temperature related to climate change. Studies relating a water temperature increase to the increase of TTX content in its vectors seem rather inconsistent, although certain studies support an increase TTX incidence associated with higher water temperatures in the case of bivalve mollusks [111].

Climate change effects will have implications for food production, food security, and food safety. In particular, the products arising from marine production systems are expected to be affected by increased occurrence of phycotoxins. This highlights the need to implement shellfish monitoring programs, especially for emerging toxins to strengthen risk management capability and to enhance consumer protection, because ensuring safe and secure seafood is important, and climate change is one of the challenges in achieving this goal (Figure 9).

## 7. Concluding Remarks

The current regulation of marine toxins can be considered a success, given the extreme difficulty for this field to make progress in research due to the scarcity of research material. This has provided a rather good level of consumer protection through the advances and the work of many groups worldwide. However, in this field, the technological progress of the analytical equipment has not been accompanied by an equivalent advance of the toxicology and mode of action studies. The main cause of this bias is that most of the research performed in the field is undertaken by ecologists, organic chemists, and analytical chemists, and only a few toxicologists. This review emphasizes the need to perform further and deeper research into toxicology and the mode of action of the toxin groups. This would allow a better use of marine toxins as drug leads, but mostly to better use the toxicological research as a needed tool for the current analytical regulation. This is rather important, since LC-MS has been set as a monopoly for phycotoxins monitoring, in opposition to the philosophical regulation of mycotoxins that are legislated based on any method that follows the requirement of minimum performance characteristics.

## Figures and Tables

**Table 2:** Toxicity equivalency factors for ciguatoxins depending on the testing assay.

Toxin	TEF EFSA [124]	TEFs Based on In Vitro Assay: Na_v_1.6 Current Peak [59]	TEFs Based on In Vitro Assay: Na_v_1.6 Shift Activation Potential [59]
P-CTX-1	1	1	1
P-CTX-2	0.3		
P-CTX-3	0.3	0.28	0.28
P-CTX-3C	0.2		
2,3-dihydroxy P-CTX-3C	0.1		
51-hydroxy P-CTX-3C	1		
P-CTX-4A	0.1	0.0016	0.1
P-CTX-4B	0.05		
C-CTX-1	0.1		
C-CTX-2	0.3		

**Table 3:** Toxicity equivalency factors for DSTs depending on the assay.

Toxin	TEF EFSA [205]	TEF FAO [70]	TEFs Based on Oral Gavage [39,40]	TEFs Based on In Vitro IC_50_ for PP2A [206]	TEFs Based on PP2A Inhibition by FAO [70]
OA	1	1	1	1	1
DTX1	1	1	1.5	1.6	1.6
DTX2	0.6	0.5	0.3	0.3	0.5

IC_50_: inhibitory concentration 50.

**Table 4:** Toxicity equivalency factors for azaspiracids depending on the assay.

Toxin	TEF EFSA [19]	TEF FAO [70]	TEFs Based on Oral Gavage [215]
AZA1	1	1	1
AZA2	1.8	0.7	0.7
AZA3	1.4	0.5	0.5
AZA6		0.7	

**Table 5:** Old spread knowledge versus updated evidence in marine phycotoxins.

	Old Facts	Current Facts
**Mechanism of Action**		
OA	PP inhibition, diarrhea caused by disruption of gap-junctions	PP inhibition in combination with neurological pathways
AZAs	Main target hERG	Remains unknown
CTX	Sodium current peak amplitude	Sodium current peak amplitude and activation shift to more negative potential
**TEFs**		
OA	OA ≈ DTX1	DTX1 > OA
STX	STX ≈ NeoSTX ≈ dcSTX	NeoSTX > STX > dcSTX
**Toxicity**		
Maitotoxins	Most potent toxins	No CFP cases reported, nor oral toxicity detected
PTXs	Causative DSP agent	Not causative of DSP, no human poisoning reported
TTX	Minimum lethal dose for humans 2 mg	EFSA proposed ARfD of 0.25 μg/kg bw
**European legislation**		
PTXs	Regulated as part of OA group of toxins	Not legislated anymore
YTX	Legal limit 1 mg YTX equivalents/kg shellfish	Legal limit 3.75 mg YTX equivalents/kg shellfish
TTX	Emerging toxins responsible for human poisoning	Detected in seafood products more frequently, non-regulated
CTX		
PLTX		
CIs	Emerging toxins	

**Table 6:** Aptamer-based biosensors for marine toxins.

Toxin	Aptamer Name	Aptamer Sequence	Sensor Technology	LOD (ng/mL)	Seafood Samples	Reference
STX	M-30f	TTG AGG GTC GCA TCC CGT GGA AAC AGG TTC ATTG	Biolayer interferometry	0.5 ng/mL	Shellfish	[388]
STX	APT^STX^	GGT ATT GAG GGT CGC ATC CCG TGG AAA CAT GTT CAT TGG GCG CAC TCC GCT TTC TGT AGA TGG CTC TAA CTC TCC TCT	Fluorescence	7.5 ng/mL	-	[385]
STX	-	TTT TTT AGG GAA GAG AAG GAC ATA TGA TGG CAC AAG GCC CAT CAA TCG GTA TAC GGG TTG ACT AGT ACA TGA CCA CTT GA	Localized surface plasmon resonance	2.5 ng/mL	Mussel	[386]
STX	STX-41	ATA GGA GTC ACG ACG ACC AGC TTT TTA CAA AAT TCT CTT TTT ACC TAT ATT ATG AAC AGA TAT GTG CGT CTA CCT CTT GA	Fluorescence	0.4 ng/mL	Clam	[389]
STX	APT^STX^	GGT ATT GAG GGT CGC ATC CCG TGG AAA CAT GTT CAT TGG GCG CAC TCC GCT TTC TGT AGA TGG CTC TAA CTC TCC TCT	Attenuated Internal reflection spectroscopic ellipsometry	10 pg/mL	Shrimp	[390]
STX	APT^STX^	GGT ATT GAG GGT CGC ATC CCG TGG AAA CAT GTT CAT TGG GCG CAC TCC GCT TTC TGT AGA TGG CTC TAA CTC TCC TCT	Electrochemistry	9 pg/mL	Mussel	[391]
DA	DA-06	ATA GGA GTC ACG ACG ACC AGA AAA ATA ATT TAA ATT TTC TAC CCA ATG CTT TTC GCA TAA TAT GTG CGT CTA CCT CTT GA	Fluorescence	0.45 ng/mL	Clam	[389]
GTX1/4	GO18-T-d	AAC CTT TGG TCG GGC AAG GTA GGT T	Biolayer interferometry	50 pg/mL	Shellfish	[387]
OA	OA34	GGT CAC CAA CAA CAG GGA GCG CTA CGC GAA GGG TCA ATG TGA CGT CAT GCG GAT GTG TGG	Electrochemistry	70 pg/mL	shellfish	[383]
OA	OA34	GGT CAC CAA CAA CAG GGA GCG CTA CGC GAA GGG TCA ATG TGA CGT CAT GCG GAT GTG TGG	Electrochemistry	1 ng/mL	-	[392]
OA	OA34	GGT CAC CAA CAA CAG GGA GCG CTA CGC GAA GGG TCA ATG TGA CGT CAT GCG GAT GTG TGG	Fluorescence	50 pg/mL	shrimp	[384]
OA	OA27-1	TGT CGA GGG AGA CGC GCA GTC GCT ACC ACC T	Colorimetric (enzyme-linked aptamer assay)	10 pg/mL	Clam	[393]
OA	OA-Apt	GGT CAC CAA CAA CAG GGA GCG CTA CGC GAA GGG TCA ATG TGA CGT CAT GCG GAT GTG TGG	Colorimetric (enzyme-linked aptamer assay)	6.4 pg/mL	Mussel	[394]
Tetrodotoxin	TTX-07	ATA GGA GTC ACG ACG ACC AGT CAA ATT TTC GTC TAC TCA ATC TTT CTG TCT TAT CTA TGT GCG TCT ACC TCT TGA	Fluorescence	0.26 pg/mL	Clam, shellfish	[395]
Palytoxin	PTX-13	GGA GGT GGT GGG GAC TTT GCT TGT ACT GGG CGC CCG GTT GAA	Biolayer interferometry	0.04 pg/mL	Mussel, clam, scallop	[396]

## Data Availability

Not applicable.
